# Loss of Wwox drives metastasis in triple-negative breast cancer by JAK2/STAT3 axis

**DOI:** 10.1038/s41467-018-05852-8

**Published:** 2018-08-28

**Authors:** Renxu Chang, Lele Song, Yi Xu, Yanjun Wu, Cheng Dai, Xinyu Wang, Xia Sun, Yingyong Hou, Wei Li, Xianbao Zhan, Lixing Zhan

**Affiliations:** 1 Key Laboratory of Nutrition, Metabolism, and Food Safety, Institute of Nutrition and Health, Shanghai Institutes for Biological Sciences, University of the Chinese Academy of Sciences, Shanghai, 200031 China; 2 Department of Pathology, Zhongshan Hospital, Fudan University, Shanghai, 200032 China; 3 Department of General Surgery, Sir Run Run Shaw Hospital, College of Medicine, Zhejiang University, Zhejiang, 310020 China; 4 Department of Oncology, Changhai Hospital, Second Military Medical University, Shanghai, 200433 China; 5 Department of Cellular and Genetic Medicine, Key Laboratory of Metabolism and Molecular Medicine, Ministry of Education, School of Basic Medical Sciences, Fudan University, Shanghai, 200032 China

## Abstract

Loss of WW domain-containing oxidoreductase (Wwox) expression has been observed in breast cancer (BC). However, its regulatory effects are largely unknown, especially in triple-negative breast cancer (TNBC). Herein, gene expression profiling revealed that JAK/STAT3 pathway was one of the most differentially modulated pathways in basal-like BC cells. The lower expression of Wwox was significantly correlated with high activation of STAT3 in basal-like cells and TNBC tissues. Overexpression of Wwox markedly inhibited proliferation and metastasis of BC cells by suppressing STAT3 activation, which is to interact with JAK2 to inhibit JAK2 and STAT3 phosphorylation. Furthermore, Wwox limited STAT3 binding to the interleukin-6 promoter, repressing expression of the IL-6 cytokine. Altogether, our data established that Wwox suppresses BC cell metastasis and proliferation by JAK2/STAT3 pathway. Targeting of Wwox with STAT3 could offer a promising therapeutic strategy for TNBC.

## Abstract (web summary)

In breast cancer, the loss of expression of WW domain-containing oxireductase (Wwox) has been observed. Here, the authors illustrate that in triple negative breast cancer models Wwox suppresses metastasis and proliferation via the JAK2/STAT3 pathway.

## Introduction

Globally, breast cancer (BC) is the most frequent malignancy and the leading cause of cancer-associated mortality in women^1,2^. Based on the presence/absence of estrogen receptor (ER), progesterone receptor (PR), and human epidermal growth factor-2 (Her2), BC patients can be classified into the following categories: luminal A, luminal B, HER2 overexpression, and triple-negative subtypes. Triple-negative breast cancer (TNBC) tumors often are more aggressive, are less sensitive to typical endocrine therapies, have a poorer prognosis, and have a higher rate of distant recurrence compared to other subtypes^3–6^. Less than 30% of patients with metastatic TNBC tumors survive more than 5 years after diagnosis^7^. Patients with TNBC are difficult to treat due to the heterogeneity of the tumors and the lack of well-defined molecular targets. Defining the unique characteristics of an individual patient’s tumors is beneficial to the development of therapeutic schemes that will be most effective for individual patients^8,9^.

The WW domain-containing oxidoreductase (Wwox) is a 46 kDa protein consisting of two N-terminal WW domains and a C-terminal short-chain dehydrogenase/reductase domain^10^, and is encoded by a locus that spans FRA16D^11,12^, one of the most active common fragile sites involved in cancer. The genomic location of the Wwox-encoding gene makes the locus susceptible to loss of heterozygosity and homozygous deletions, either of which results in reduced gene expression^13^. Loss of Wwox expression has been observed in cancers of many organs, including breast, lung, esophageal, and gastric carcinomas^11,14–18^. Loss of *Wwox* heterozygosity has been observed in 70% or more of the pre-invasive stages of BC samples^19^. The level of Wwox expression was shown to correlate with ER and PR status, such that expression of Wwox is higher in ER- and PR-positive tumors than in tumors that are negative for these receptors^14,20^. Additionally, a large cohort study of BC used immunohistochemical (IHC) assays to demonstrate that TNBC exhibits more frequent loss of Wwox expression^21^. Wwox has been proven to be involved in a variety of cellular processes, including cell differentiation, apoptosis, and growth, and these effects are mediated through interactions with various proteins such as p73^22^, AP-2γ^23^, and ErbB4^24,25^. However, the regulatory effects of Wwox have not been well characterized, especially in TNBC. In the present study, we found that Wwox affects the IL-6/JAK2/STAT3 (interleukin-6/Janus kinase-2/signal transducer and activator of transcription-3) axis, inhibiting cancer cell growth and metastasis in a STAT3-dependent manner.

## Results

### Wwox is negatively correlated with STAT3 activity in BC

**Fig. 1:**
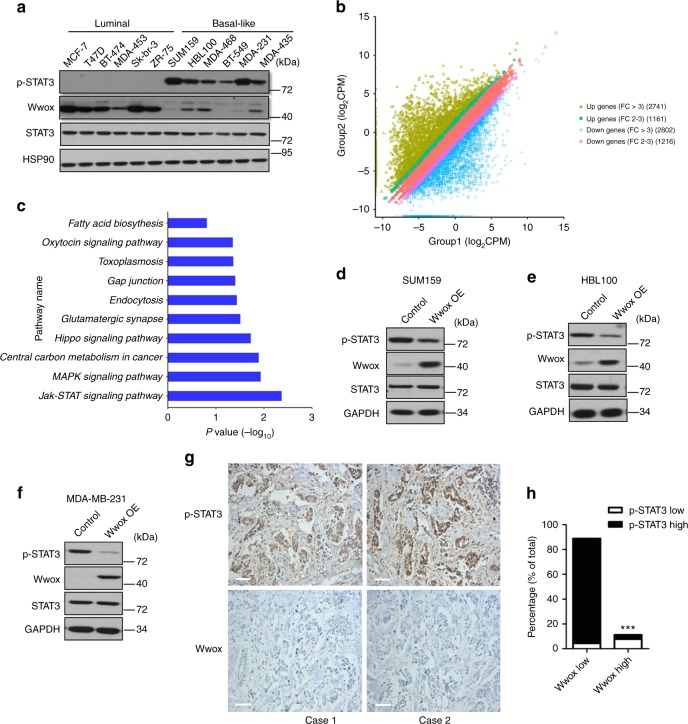
Wwox and p-STAT3 exhibit converse patterns of expression in breast cancer (BC) cells. **a** Western blotting was performed to detect p-STAT3 and Wwox levels in 12 human BC cell lines. The protein levels of p-STAT3 were detected. **b** 2D plots of total RNA-seq genes in Group 1 luminal cells (MCF-7, T47D, BT-474) and Group 2 basal cells (SUM159, HBL100, BT-549). Up- and downregulated genes with fold change ≥2.0 are highlighted. **c** KEGG pathway analysis of up-regulated genes in basal cells compared with luminal cells. **d**–**f** Western blot analysis of Wwox and p-STAT3 expression in SUM159 (**d**), HBL100 (**e**), and MDA-MB-231 cells (**f**) stably transfected with control (CT) or Wwox-encoding vectors. **g**, **h** IHC determination of Wwox and p-STAT3 expression in 90 human triple-negative BCs. Representative IHC staining images of Wwox and p-STAT3 (**g**); results are tabulated as shown (**h**); ***p < 0.001, χ^2^ test, scale bar, 100 μm

Firstly, we characterized the level of Wwox protein in BC cells, and Wwox protein was expressed at much lower levels in basal-like cells than in luminal cells (Fig. 1a). RNA sequencing (RNA-seq) experiments were performed to identify potential differences in gene expression levels between these two BC subtypes. A total of 7920 protein-encoding genes appeared to be deregulated in basal cells (Fig. 1b). Analysis using gene ontology (GO) and Kyoto Encyclopedia of Genes and Genomes (KEGG) pathway analyses (Supplementary Fig. 1a, b, Fig. 1c) revealed that the JAK/STAT3 pathway was one of the most differentially modulated canonical pathways in basal BC cells. We therefore examined the phosphorylation state of STAT3 in different BC cells. We observed that STAT3 was persistently phosphorylated in basal BC cells; the expression of Wwox was detected only at very low levels in these cells. In contrast, Wwox was highly expressed in luminal BC cells that exhibited very low levels of phosphorylated STAT3 (p-STAT3; Fig. 1a).

To determine whether increased expression of Wwox might inhibit STAT3 activation, we overexpressed Wwox into basal-like cells (SUM159, HBL100, and MDA-MB-231), and found that exogenous Wwox suppressed STAT3 activation (Fig. 1d–f). In contrast, STAT3 was activated when we knocked down Wwox in luminal cells (T47D, SKBR-3, MCF-7, and MCF-10A) (Supplementary Fig. 1c–f). Notably, IHC analyses revealed that Wwox expression was rarely detected in virtually all of the 90 TNBC samples (Fig. 1g, h); however, STAT3 was dramatically activated in these TNBC samples. We also examined the expression patterns of Wwox and p-STAT3 in 30 BC tissues and their paired adjacent normal tissues. IHC analyses showed that the low expression of Wwox correlated with increased STAT3 activation in BC tissues (Supplementary Fig. 1g, h). Taken together, these results suggested that the expression of the Wwox protein inversely correlates with the activation of STAT3, implying that Wwox may negatively regulate STAT3 activation.

### Wwox inhibits tumor growth by suppressing STAT3 activity

**Fig. 2:**
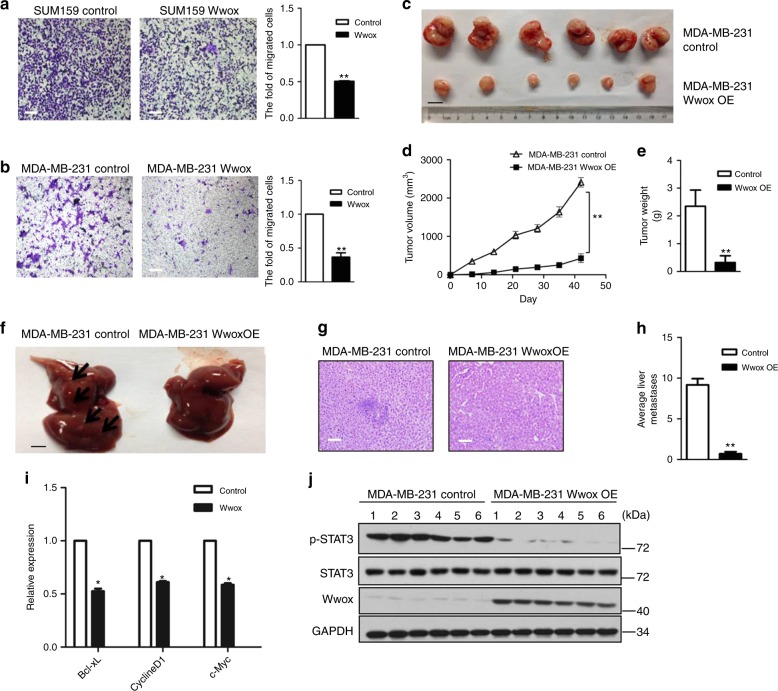
Wwox inhibits metastasis and tumor proliferation. **a**, **b** Transwell migration assays were performed with SUM159 and MDA-MB-231 cells stably transfected with control or Wwox-encoding vectors. Representative images of migrated SUM159 (**a**) and MDA-MB-231 (**b**). Quantitative results are respectively illustrated for migration in **a**, **b**. Data represent the mean ± SD (*n* = 3) from three independent experiments; **p < 0.01, Student’s *t*-test. Scale bar, 100 μm. **c**–**e** Wwox inhibits MDA-MB-231 proliferation in vivo. Cells of mock-transfected MDA-MB-231 and Wwox-overexpressing MDA-MB-231(Wwox OE) lines were orthotopically transplanted into the mice mammary fat pad. Tumor sizes were monitored over a period of 21 days (**d**). At necropsy, tumors were harvested, photographed (**c**), and weighed. Results are presented as mean ± SD (*n* = 6) of calculated tumor weight (**e**); **p < 0.01, Student’s *t*-test. Scale bar, 1 cm. **f**–**h** Liver tissues were photographed, fixed, and stained with hematoxylin and eosin (H&E) (**g**); black arrows indicate the liver metastatic lesions (**f**). The number of metastatic lesions in each specimen was counted (**h**). Results are presented as mean ± SD (*n* = 6); **p < 0.01, Student’s *t*-test. (**f**), Scale bar, 1 cm, (**g**) scale bar: 100 μm. **i** Expression of STAT3-targeted genes was examined in indicated MDA-MB-231 cells. Results shown are mean ± SD from three repeats; *p < 0.05. **j** Western blot analysis of the levels of p-STAT3 in MDA-MB-231 Wwox-overexpressing tumors and control tumors from orthotopic xenograft transplantation experiments

To characterize the effect of Wwox deficiency on the tumorigenicity of BC cells, we employed several cell culture models.The transwell migration assay revealed that cellular migration was attenuated upon overexpression of Wwox in SUM159, MDA-MB-231 (Fig. 2a, b), and HBL100 cells (Supplementary Fig. 2a, b). Conversely, knockdown of Wwox in MCF-10A significantly increased cell mobility compared with control cells (Supplementary Fig. 2c, d). In separate experiments, we used three-dimensional (3D) culture conditions; this cell culture technique recreates the architecture of epithelial tissue growth in vitro. In contrast to control cells, *Wwox*-silenced cells formed larger spheroid-like structures. Moreover, further inhibition of STAT3 activation in MCF-10A shWwox cells suppressed the growth of these spheroid-like structures (Supplementary Fig. 2e). These results suggested that Wwox can inhibit cell migration in vitro.

Next we investigated the effect of Wwox on BC proliferation and metastasis in vivo by utilizing orthotopic xenograft transplantation into the mouse mammary fat pad. We found that animals implanted with Wwox-overexpressing cells exhibited dramatically reduced tumor size, tumor growth rate, tumor weight, and number of liver metastases compared with control animals (Fig. 2c–h). As expected, the expression of STAT3-regulated genes was reduced in Wwox-overexpressed cells (Fig. 2i). In addition, the p-STAT3 levels were significantly decreased in Wwox-expressing MDA-MB-231 tumors (Fig. 2j). Thus, both in vitro and in vivo assays demonstrated that Wwox inhibits metastasis of BC cells.

To obtain direct evidence that Wwox inhibits STAT3 phosphorylation during tumor growth, we overexpressed either STAT3C, a constitutively active form of STAT3^26^, or v-SRC, a protein widely shown to transform NIH3T3 cells by inducing constitutive STAT3 phosphorylation. Overexpression of Wwox dramatically decreased the level of p-STAT3 in the STAT3C- or v-SRC-transformed cells (Supplementary Fig. 3a). Migration and p-STAT3-dependent colony formation of the transformed cells was attenuated when Wwox was overexpressed (Supplementary Fig. 3b-e). In the xenograft transplantation tumor model, these cell lines yielded smaller tumors when Wwox was overexpressed compared with control cells. Both tumor growth rate and tumor weight were reduced in transformed cells transfected with Wwox (Supplementary Fig. 3f–k).

STAT3 has been shown to be constitutively phosphorylated in B16 murine melanoma cells^27,28^. Intriguingly, the level of p-STAT3 was downregulated when Wwox was expressed in B16 cells (Supplementary Fig. 4a). To examine the effect of Wwox on cell transformation, a soft agar colony formation assay was performed. B16 cells with Wwox overexpression exhibited a decreased ability to form colonies (Supplementary Fig. 4b, c). We next investigated the tumorigenic capacity of Wwox-overexpressing B16 cells. Tumor size, tumor growth rate, and tumor weight were reduced in mice harboring B16 cells transfected with Wwox (Supplementary Fig. 4d–f). We also investigated the effect of Wwox on metastasis in pulmonary metastasis model. The results showed that Wwox impeded metastasis (Supplementary Fig. 4g–i). Taken together, our data suggested that Wwox negatively regulates migration in vitro and suppresses metastasis in vivo by targeting p-STAT3.

### Wwox interacts with STAT3

**Fig. 3:**
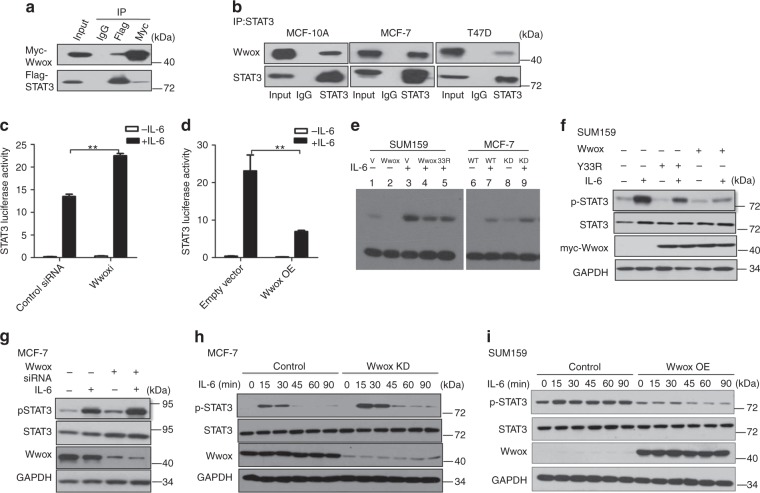
Wwox inhibits STAT3 phosphorylation. **a** Co-immunoprecipitation of Wwox and STAT3 shows Wwox–STAT3 interaction in vivo. 293T cells were transfected with plasmids expressing Myc-tagged full-length Wwox or Flag-tagged full-length STAT3, as indicated. Whole-cell lysates were used in immunoprecipitation (IP) with anti-Myc antibody or anti-Flag M2 antibody. **b** Endogenous Wwox and STAT3 interact in vivo. Lysates of MCF-10A, MCF-7, and T47D cells were subjected to IP with anti-STAT3 antibodies. **c**, **d** Wwox inhibits STAT3 transcriptional activity. MCF-7 cells were co-transfected with APRE-luciferase reporter and *Renilla* luciferase reporter, in backgrounds providing depletion (via RNAi) of Wwox (Wwoxi) (**c**) or overexpression of Wwox (Wwox). **d** Relative luciferase activity data are presented as mean ± SD from three independent experiments; **p < 0.01. **e**–**g** Wwox decreases the DNA-binding ability of STAT3. EMSA assays were performed using a biotin-labeled high-affinity binding site for STAT3. SUM159 cells were transfected with empty vector (V) or construct encoding Wwox or Wwox-Y33R mutant; MCF-7 cells were knocked down (KD) with Wwox siRNA (**e**). SUM159 or MCF-7 cells were treated with or without IL-6 for 30 min. Levels of the proteins used in EMSA are shown together with the p-STAT3 level in **f**, **g**. **h**, **i** Wwox suppresses STAT3 phoshorylation. Cells were treated with IL-6 for the indicated time intervals, using MCF-7 cells in which Wwox was knocked down (Wwox KD) or in SUM159 cells overexpressing Wwox (Wwox OE). Levels of phosphorylated STAT3 (p-STAT3) and total STAT3 were determined

Wwox has been defined as a partner of multiple transcription factors, including p73^22^ and Jun^29^. We therefore hypothesized that there might be an interaction between Wwox and STAT3, a possibility that we investigated via immunoprecipitation (IP) experiments (Fig. 3a). The interaction of endogenous Wwox and STAT3 was confirmed (Fig. 3b). Co-IP results additionally revealed that Wwox interacted with STAT5 and with STAT1 (Supplementary Fig. 5a, b). It appeared that the association between Wwox and STAT3 occurred via the WW1 domain of Wwox (Supplementary Fig. 5c, d) and the coiled-coil domain of STAT3 (Supplementary Fig. 5e, f).

We further investigated interactions between Wwox and different forms of STAT3. The results revealed that Wwox interacted with STAT3-Y705F, which is an inactive form of STAT3 that localizes primarily to the cytoplasm. In addition, Wwox retained affinity for STAT3C, a constitutively activated and dimeric form of STAT3. A derivative of STAT3C that was further mutated at Y705 interacted more weakly with Wwox than did wild-type STAT3 (Supplementary Fig. 5g).

In the Wwox protein, tyrosine 33 (Y33), located in the first WW domain, was previously reported to abolish the Wwox interaction with p73^22^. We found that a Y33R mutant Wwox protein, in which Tyr33 is replaced with an Arg residue, was still able to interact with STAT3 (Supplementary Fig. 5h). IP experiments using deletion mutants of STAT3 showed that the coiled-coil domain of STAT3 is responsible for the interaction with Wwox (Supplementary Fig. 5i). Collectively, these results demonstrated that Wwox interacts with STAT3 via sequence-specific recognition.

### Wwox impedes STAT3 phosphorylation

To address whether Wwox affects STAT3 transcriptional activity, we performed luciferase reporter assays using the STAT3-specific binding element APRE. IL-6-induced STAT3 transcriptional activity was inhibited by transient overexpression of Wwox, but was enhanced by knockdown of Wwox using short interfering RNA (siRNA; Fig. 3c, d, Supplementary Fig. 6a, b). Furthermore, the Wwox effect of IL-6 on APRE-driven transcriptional activity was dose dependent (Supplementary Fig. 6c). Therefore, we concluded that Wwox inhibits the transcriptional activity of STAT3.

To clarify the molecular mechanism of Wwox inhibition of STAT3 activity, we performed an electrophoretic mobility shift assay (EMSA) to examine whether Wwox affected STAT3 DNA-binding ability. Under standard conditions, incubation of cells with IL-6 results in DNA binding by STAT3. However, exogenous expression of Wwox attenuated IL-6-induced DNA binding by STAT3 (Fig. 3e, Supplementary Fig. 6d). Conversely, depletion of Wwox cells resulted in enhanced STAT3 DNA-binding activity (Fig. 3e, Supplementary Fig. 6d). Thus, Wwox inhibits STAT3 transcriptional activity by blocking STAT3 DNA-binding activity.

We also observed that IL-6-induced accumulation of p-STAT3 was attenuated when Wwox was overexpressed in SUM159 cells (Fig. 3f). Conversely, IL-6-induced accumulation of p-STAT3 was potentiated in MCF-7 (Fig. 3g) and MCF-10A cells (Supplementary Fig. 6e) depleted for Wwox. Notably, Wwox protein levels were unchanged when STAT3 was depleted by siRNAs (Supplementary Fig. 6f). Neither the Wwox protein levels nor STAT3 activation were changed when ER was overexpressed (Supplementary Fig. 6g)

To distinguish whether Wwox inhibits the phosphorylation of STAT3 or accelerates STAT3 dephosphorylation, we employed cytokine stimulation assays. Western blot analysis showed that the maximum levels of p-STAT3 increased following incubation of Wwox knocked-down MCF-7 cells with IL-6 for increasing time intervals (Fig. 3h). In contrast, the maximum levels of IL-6-stimulated p-STAT3 were decreased in SUM159 (Fig. 3i) or in B16 (Supplementary Fig. 6h) cells with Wwox overexpression. We further tested the mechanism of Wwox effect on STAT3 activation using cytokine addition–withdrawal assays; p-STAT3 levels did not differ between cells that were depleted for Wwox or overexpressing Wwox in comparison to control cells (Supplementary Fig. 6i, j). Taken together, these data indicated that the effect of Wwox on the accumulation of p-STAT3 is mediated via STAT3 phosphorylation.

### Wwox inhibits JAK2 phosphorylation and JAK2 binding to STAT3

**Fig. 4:**
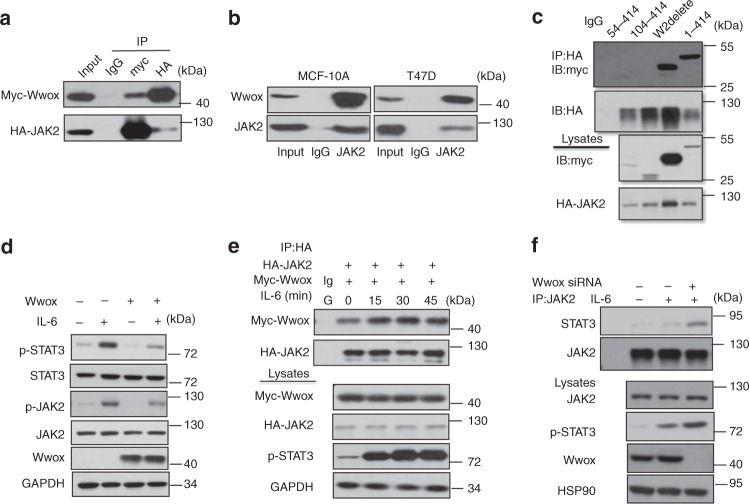
Wwox inhibits the interaction of JAK2 and STAT3. **a** Co-immunoprecipitation of Wwox and JAK2 shows Wwox–JAK2 interaction. 293T cells were transfected with plasmids expressing Myc-tagged full-length Wwox or HA-tagged full-length JAK2, as indicated. Whole-cell lysates were used in immunoprecipitation (IP) with anti-Myc antibody or anti-HA antibody. **b** Endogenous Wwox and JAK2 interact in vivo. Lysates of MCF-10A and T47D cells were subjected to IP with anti-JAK2 antibodies. **c** Wwox interacts with JAK2 mainly through the WW1 domain. IP and immunoblot (IB) of cell lysates from 293T cells expressing HA-tagged STAT3 and Myc-tagged Wwox or Myc-tagged truncated Wwox proteins (illustrated in Supplementary Fig. 5). Whole-cell lysates were immunoprecipitated with anti-HA and immunoblotted with anti-Myc antibody. **d** Wwox reduces p-STAT3 and p-JAK2 levels. MDA-MB-231 cells were transfected with Wwox plasmids and were treated with IL-6 for 30 min. The whole-cell lysates were immunoblotted with the indicated antibodies. **e** Interaction pattern of Wwox and JAK2 under IL-6 stimulation. IP and IB of cell lysates from 293T cells expressing HA-tagged JAK2 and Myc-tagged Wwox; cells were treated with IL-6 for the indicated time spans. **f** Wwox inhibits the interaction of JAK2 with STAT3. MCF-7 cells were transfected with Wwox siRNA, along with IL-6 stimulation; cell lysates were subjected to IP and IB analyses

The JAK2/STAT3 pathway has been widely studied in BC and other cancer types^30,31^. Using co-IP, we confirmed that Wwox interacts with JAK2 (Fig. 4a, b). In addition, Wwox was also shown to interact with other members of the JAK kinase family (Supplementary Fig. 7a, b). Using a series of plasmids encoding truncated JAK2 and Wwox proteins, the association between JAK2 and Wwox occurs via the WW1 domain of Wwox and the TycKc domain of JAK2 (Fig. 4c, Supplementary Fig. 7c, d). We next tested whether Wwox overexpression induced JAK2 activation. The results revealed that Wwox attenuated IL-6 induced JAK2 phosphorylation (Fig. 4d). Nonetheless, the interaction between JAK2 and Wwox was enhanced when cells were stimulated by exposure to IL-6 for different time intervals (Fig. 4e).

To further address whether Wwox suppresses the association of JAK2 with STAT3, we examined the interaction between JAK2 and STAT3 in cells performing knockdown of Wwox. The interaction between JAK2 and STAT3 was increased upon downregulation of Wwox (Fig. 4f). We also found that the interaction between JAK2 and STAT3 was attenuated upon overexpression of Wwox (Supplementary Fig. 7e). In this context, we identified several potential phosphorylation sites in the WW1 domain of Wwox and found that the interaction between JAK2 and Wwox was decreased when residue S14 or T49 of Wwox was mutated (Supplementary Fig. 7f). Notably, the association of JAK2 with STAT3 was restored when Wwox S14A or T49A was overexpressed (Supplementary Fig. 7g), in contrast to the effect seen upon overexpression of wild-type Wwox. Overexpression of S14- or T49-mutant Wwox proteins did not inhibit the IL-6-mediated induction of STAT3 transcriptional activity (Supplementary Fig. 7h). Together, these results indicated that Wwox decreases STAT3 activation by inhibiting JAK2 phosphorylation.

### Wwox inhibits STAT3-mediated induction of IL-6

**Fig. 5:**
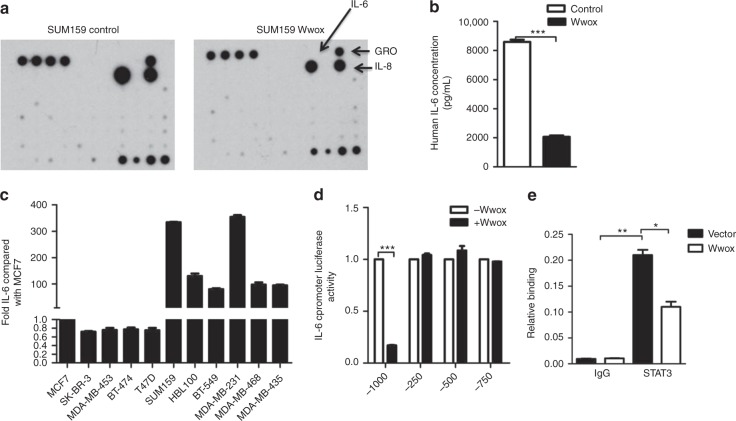
Wwox inhibits IL-6 induction. **a** Cytokine array analysis of conditioned medium (CM) from SUM159 cell culture. Equal numbers of SUM159 cells transfected with control empty vector (159 control) or with Wwox overexpression construct (159 Wwox) were seeded on culture plates and incubated in DMEM supplemented with 10% FBS for 24 h at 37 °C to allow cell attachment; culture medium was then switched to DMEM without serum. After incubation for 48 h, CM was collected and centrifuged at 2000 × *g* for 10 min at 4 °C to remove cell debris. The resulting supernatant was used for the experiment. **b** ELISA quantification of IL-6 production in the same CMs analyzed by the cytokine array in **a**. **c** IL-6 production by various luminal breast cancer (BC) cells and basal-like BC cells analyzed by ELISA compared with IL-6 production by MCF-7 cells. **d** Wild-type (−1000 to +1 bp with respect to the transcription start site) and truncated *IL-6* promoter constructs were co-transfected with a vector encoding Wwox, and the luciferase activity was determined. **e** 293T cells were transfected with Wwox plasmids, and cultures were analyzed for STAT3 binding to the *IL-6* promoter. Data represent the mean ± SD (*n* = 3) from three independent experiments; *p < 0.05, **p < 0.01, ***p < 0.001, Student’s *t*-test

STAT3 functions as a transcription factor, and so we hypothesized that Wwox may have an effect on STAT3-mediated transcriptional activity function. We therefore investigated the potential role of Wwox in the secretion of the cytokines by basal BC cells. The cytokine profiles of conditioned medium were analyzed using Cytokine Antibody Array. The levels of secreted IL-6 exhibited the largest decrease in CM from SUM159 Wwox-overexpressing cells (Fig. 5a). We further confirmed that the levels of both *IL-6* messenger RNA (mRNA) and secreted IL-6 protein were attenuated in the Wwox-transfected SUM159 cells (Fig. 5b, Supplementary Fig. 8a). The decreased levels of secreted IL-6 protein were confirmed (using enzyme-linked immunosorbent assay (ELISA)) in Wwox-transfected MDA-MB-231, HBL100, and BT-549 cells (Supplementary Fig. 8b-d); similarly, the levels of *IL-6* mRNA were decreased in Wwox-transfected MDA-MB-231 and BT-549 cells (Supplementary Fig. 8e, f).

STAT3 activation is known to be required for IL-6 production, and the JAK2/STAT3, nuclear factor (NF)-κB, and p38 signaling pathways have been shown to promote IL-6 induction in human cells^31–34^. We found that *IL-6* mRNA accumulates to higher levels in basal cells than in luminal cells, and that *IL-6* mRNA expression is negatively correlated with *Wwox* expression (Supplementary Fig. 8g). These results suggested that Wwox suppresses *IL-6* mRNA expression and IL-6 protein production in TNBC.

To determine whether Wwox regulates *IL-6* transcription, we assayed *IL-6* transcription using a luciferase-encoding reporter under the control of the human *IL-6* promoter. We observed that Wwox overexpression led to decreased *IL-6* promoter-driven luciferase production (Fig. 5d). We speculated that STAT3 might mediate the induction of IL-6 expression via the partial *IL-6* promoter on loss of Wwox in BC cells. Chromatin immunoprecipitation (ChIP) confirmed that STAT3 directly bound to the *IL-6* promoter. Furthermore, forced expression of Wwox in STAT3-overexpressing cells prevented STAT3 recruitment at the truncated *IL-6* promoter (Fig. 5e). Taken together, these results demonstrated that Wwox inhibits STAT3-mediated transcription from the *IL-6* promoter.

### The level of Wwox has prognostic implications in BC

**Fig. 6:**
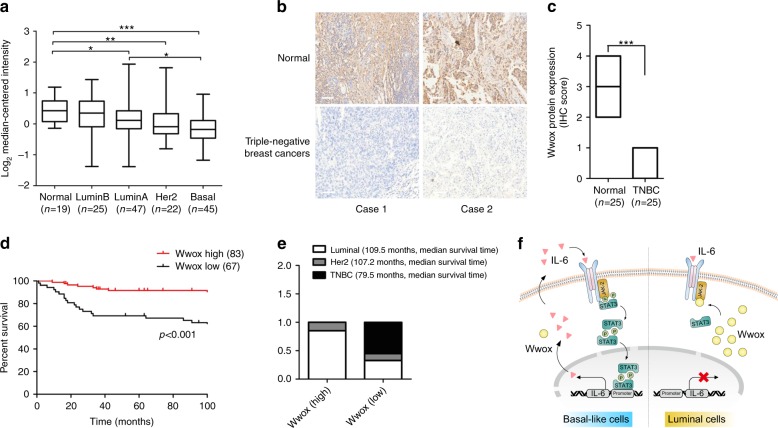
The levels of Wwox expression in breast cancer (BC) have prognostic implications. **a** The mRNA expression profiles of *Wwox* in normal breast tissues and in different molecular subtypes of BC; *p *<* 0.05, **p *<* 0.01, ***p *<* 0.001. Student’s *t*-test. **b**, **c** IHC staining for Wwox in adjacent normal and paired triple-negative BCs (TNBCs) (**b**). Box plots of Wwox protein expression assessed by blinded IHC analyses of 25 normal and paired TNBC tissues (**c**); ***p < 0.001, Student’s *t*-test. Scale bar, 100 μm. **d** Kaplan–Meier curves for overall survival in 150 human BC patients classified by relative (high or low) immune signal for Wwox protein levels. The log-rank (Mantel–Cox) test *p* value reflects the significance of the correlation between Wwox positivity and longer survival outcomes; ***p < 0.001, log-rank test. **e** The percentage of Wwox protein levels in different subtypes of 150 human BC samples. The definitions of 'high' and 'low' are given in the Methods section. **f** A model of the regulation of STAT3 activity by Wwox in BC. Wwox inhibits JAK2 phosphorylation and attenuates the interaction between JAK2 and STAT3, suppressing STAT3 phosphorylation and inhibiting IL-6 production

We examined Wwox expression levels in clinical specimens. In silico analyses^35^ showed that *Wwox* mRNA expression levels were much lower in basal BCs compared to those in normal tissues (Fig. 6a), and that *Wwox* mRNA levels in basal BCs were even significantly lower than those in luminal BCs (Fig. 6a). In addition, in silico analyses of three other independent datasets, obtained from Oncomine^36–38^, revealed a very similar result in breast carcinoma patient samples and paired normal tissues (Supplementary Fig. 9a–c). Furthermore, we employed a scoring approach based on the annotation of the IHC images. Specifically, IHC staining of 30 paired normal and BC tissues found that the protein levels of Wwox were lower in the BC specimens (Supplementary Fig. 9d, e). IHC staining of 25 paired normal and TNBC tissues revealed that Wwox protein levels were dramatically lower in the TNBC specimens (Fig. 6b, c).

To investigate the correlation of Wwox expression with clinical features of BCs, we examined the protein expression levels of Wwox in 150 BC patient tissues by IHC. Kaplan–Meier analysis of specimens from human patients with BC revealed that patients harboring tumors with high Wwox protein levels (*n* = 83, median survival time = 116 months) had longer overall survival times than did patients harboring tumors with low Wwox protein levels (*n* = 67, median survival time = 82 months; *p* < 0.001) (Fig. 6d). As expected, all the TNBC cases are present Wwox low expression pattern in this cohort. We note that all the median survival time of TNBC patients is much shorter than other subtypes of BC patients (Fig. 6e). Moreover, the decreased level of Wwox protein was associated with larger tumor size and higher tumor grade level in patients (Supplementary Table 1). We further utilized the databases from the online Kaplan–Meier plotter website to perform in silico analysis of *Wwox* mRNA expression data^39^. The frequency of relapse-free survival (RFS) was worse in patients with low *Wwox* expression levels (*n* = 1977) than in patients with high *Wwox* expression levels (*n* = 1974, respectively; *p* < 0.001) (Supplementary Fig. 8f). These clinical findings indicated that low *Wwox* expression level may be prognostic for the malignant progression of BC.

A proposed model for the Wwox regulatory role is summarized schematically in Fig. 6f. As shown in this model, luminal cells exhibit abundant Wwox expression. During IL-6 stimulation, Wwox is capable of competitive interaction with JAK2, leading to the inhibition JAK2 and STAT3 phosphorylation. In contrast, basal cells exhibit low levels of Wwox, permitting the activation of JAK2, the interaction of JAK2 with STAT3, and STAT3 phosphorylation. The p-STAT3 translocates into the nucleus, where p-STAT3 *trans*-activates *IL-6* transcription; the resulting IL-6 secretion subsequently may promote further constitutive STAT3 activation.

## Discussion

As reported here, we demonstrated that the level of Wwox protein negatively correlates with STAT3 activation, not only in BC cells, but also in clinical BC specimens. Additionally, we showed that overexpression of Wwox inhibits STAT3 activity in basal BC cells. Moreover, we demonstrated that Wwox inhibits tumor growth and metastasis by basal-like BC cells.. We further demonstrated that Wwox inhibits JAK2 phosphorylation and impedes the association of JAK2 with STAT3, thereby inhibiting STAT3 phosphorylation. In addition, Wwox suppresses *IL-6* mRNA expression and IL-6 production by inhibiting the binding of STAT3 at the *IL-6* promoter. Furthermore, we showed that patients harboring tumors with higher Wwox protein levels exhibited longer RFS and overall survival times. In summary, our studies suggested that Wwox is an inhibitor of the malignant progression of BC, and that this regulatory effect is mediated through the IL-6/JAK2/STAT3 axis.

Many studies have shown that *Wwox* is frequently deleted or altered in multiple malignant cancers^40^. Decreased Wwox expression is frequently observed in TNBC and invasive BC subtypes that are associated with high local recurrence rates, lack of effective target therapies, distant metastases, and poor disease-free survival^15,20,21,41^. Previous studies have shown that Wwox functions predominantly through its first WW domain, which physically interacts with PPxY-containing proteins, including AP-2γ^23^ and SMAD3^42^, sequestering the PPxY-containing proteins in the cytoplasm to suppress their transcriptional functions. In the present study, we found that the low expression of Wwox is significantly correlated with highly activated STAT3 in basal cancer cells and in TNBC tissues. Decreased Wwox expression is associated with the triple-negative subtype and a poor disease-free survival rate for BC patients. Wwox interacts with STAT3 or JAK2 via Wwox first WW domain, and this interaction is independent of PPxY motifs. Phosphorylation of the Y33 residue of Wwox has been shown to enhance recognition of the PPxY motif and to promote the WW–PPxY interaction^22,23,25^. However, in the present work, the interaction of Wwox with STAT3 was not affected when the Y33 residue of Wwox was mutated. Intriguingly, we identified some potential phosphorylation sites in the Wwox protein sequence and showed that mutation of these residues affect Wwox interaction with JAK2. These candidate sites included Wwox S14, a residue that has previously been shown to be important for protein–protein interactions^43^. However, we did not identify a cognate kinase responsible for phosphorylation of the Wwox S14 residue. We did not find the evidence that ER can directly regulate Wwox–STAT3 signaling pathway. However, it was reported that *S*-glutathionylation of cysteine residues in ER protein influences the cellular consequences and cytotoxic effects in dendritic cells^44^. Further investigations will be needed to explore the role that ERα plays in the Wwox function and the role of Wwox phosphorylation in BC.

Persistent STAT3 activation has frequently been linked to more malignant cancer behaviors, including proliferation, invasion, and metastasis^45^. Aberrant JAK2/STAT3 signaling has been detected in a variety of tumor types, indicating that STAT3 inhibitors might to be widely effective as anticancer therapies^30,31^. STAT3 activity is tightly regulated by phosphorylation activators and phosphorylation inhibitors. The protein inhibitors of activated STATs and the suppressors of cytokine signaling proteins (SOCSs) act as negative feedback regulators to prevent further JAK/STAT signal activation^46,47^. Among these negative regulators, SOCS3 is widely recognized for its ability to attenuate IL-6-induced STAT3 activation^48,49^. The accumulation of SOCS3 protein has been shown to enhance SOCS3 association with JAK2, which then promotes the ubiquitination and degradation of JAK2, resulting in a loss of STAT3 phosphorylation and function^50^, indicating that Wwox and SOCS3 negatively regulate STAT3 activity by distinct regulatory mechanisms.

In the previous study, conditional deletion of Wwox in the mouse mammary gland does not result in tumorigenicity, although activation of STAT3 in the mouse mammary gland was observed^51^. However, the precise mechanisms linking STAT3 signaling to the function of *Wwox* in tumorigenesis have not been explored. In the present study, we demonstrated that Wwox impedes the association of JAK2 with STAT3, thereby inhibiting STAT3 phosphorylation and p-STAT3-dependent cancer cell growth in vivo. Using NIH3T3 fibroblasts with STAT3 constitutive activation, we further proved that Wwox ability to suppress in vivo tumor growth depends on STAT3 activation. Our study provides new insights into how Wwox serves as an important negative regulator by suppressing STAT3 activation in the pathogenesis of BC.

Increased cytokine receptor signaling, such as that transduced by the IL-6 receptor, has been implicated as one of the reasons for prolonged STAT3 activation, given that such cytokines are continuously released in autocrine or paracrine manners^52,53^. IL-6 levels are dramatically increased in metastatic diseases, and elevated levels of serum IL-6 are associated with poor disease outcome and prognosis in BC patients^54,55^. Additionally, depletion of IL-6 has been shown to yield reductions in tumor burden and metastasis in experimental models^56–60^. Our data suggested that persistently activated STAT3 contributes to IL-6 production in basal cells. However, when Wwox protein levels are elevated in luminal cells, STAT3 phosphorylation is decreased, suppressing *IL-6* transcription. Other studies have reported that STAT3 can bind directly to the *IL-6* promoter in human cells^31,61^. However, we found that STAT3 binds to the *IL-6* promoter at a site distinct from that identified in the previous studies. Given that the NF-κB and p38 signaling pathways are known to promote IL-6 induction^33,34^, we cannot exclude the possibility that the Wwox induction of IL-6 expression in our system depends on one or both of these pathways. In addition, we observed Wwox-mediated induction of IL-8 and growth-regulated oncogene (GRO) in SUM159. Further investigations will be needed to address the potential effects of IL-8 and GRO on tumor growth.

In conclusion, our analyses have revealed that Wwox inhibits STAT3 phosphorylation, thereby serving as a potential suppressor in the progression and metastasis of BC. Notably, our data may provide a novel perspective on the role of Wwox in the progress of TNBC. These findings are expected to contribute to our understanding of the function of Wwox in tumor development, while also defining Wwox as a promising therapeutic target for the treatment of BC.

## Methods

### Plasmids and constructs

The Wwox and Wwox-Y33R plasmids were kindly provided by Dr. Rami I. Aqeilan (The Hebrew University, Israel).The Flag-STAT3, Flag-STAT5, HA-STAT1, and STAT3 luciferase reporter plasmids were generously provided by Dr. Y. Eugene Chin (IHS, China). HA-JAK2 was kindly provided by Dr. Claude Haan (University of Luxembourg, Luxembourg). PCMV-Myc-JAK1 and PCMV-HA-JAK3 were obtained by PCR amplification from complementary DNA (cDNA). The wild-type and mutant IL-6 promoters (−1000 to +1 bp) were constructed from synthetic oligonucleotides and cloned into the pGL 3.0 basic vector (Promega) using *Kpn*I and *Xho*I restriction sites. A series of myc-wwox and flag-stat3 deletion constructs, along with Wwox and STAT3 mutant plasmids, were generated by point mutation Site-directed Gene Mutagenesis Kit (Beyotime) according to the manufacturers’ protocols. Plasmid constructions and PCR were performed using standard molecular biology techniques.

RNA interference (RNAi) sequences were designed using the short hairpin RNA (shRNA) library of the RNAi Codex (codex.cshl.edu). The Wwox short hairpin RNAi, which consists of a 116-base oligonucleotide with flanking sites, was synthesized and cloned into the PLKO(Puro) retroviral expression vector (Addgene) using flanking *Age*I and *Eco*RI restriction sites. The sequences were as follows:

Wwox-sh1 sense: CCATACGGATGGGAACAAGAA

Wwox-sh1 antisense: TTCTTGTTCCCATCCGTATGG

Wwox-sh2 sense: GGCGTTTACTGTGGATGATAA

Wwox-sh2 sense: GGCGTTTACTGTGGATGATAA

Wwox-sh2 antisense: TTATCATCCACAGTAAACGCC

Wwox-sh3 sense: CCTTTGCTAATGCTATGCAAA

Wwox-sh3 antisense: TTTGCATAGCATTAGCAAAGG.

Stable Wwox overexpression cell lines were generated using the retroviral vector pbabe-Wwox, which encodes full-length Wwox. Stable cell lines of STAT3C or v-SRC were established using retroviral vector Pmscv-GFP encoding full-length STAT3C or v-SRC. Lentiviral or retroviral virus preparation, infection, and selection were performed according to the respective vector’s technical manual.

### Antibodies and reagents

Antibodies and reagents were obtained as follows: anti-Wwox (sc-373846, WB, 1:1000; IHC, 1:100) antibody, STAT3 inhibitor Stattic, and all siRNAs from Santa Cruz Biotechnology; anti-STAT3 (9139S, western blot (WB), 1:1000; IP, 1:400), anti-p-STAT3 (9138S, WB, 1:1000; 9145S, IHC, 1:200), anti-JAK2 (3230S, WB, 1:1000; IP,1:200), anti-p-JAK2 (3771S, WB, 1:1000), anti-Myc (2272S, WB, 1:1000), and anti-HA (3724S, WB, 1:1000) primary antibodies and horseradish peroxidase (HRP)-conjugated secondary antibodies (7074S, 7076S, WB, 1:2000) from Cell Signaling Technology; anti-GAPDH (M2006M, WB, 1:5000) and anti-β-actin (M2011M, WB, 1:5000) antibodies from Abmart Company; anti-FLAG (SAB1306078, WB, 1:5000) antibody from Sigma-Aldrich; human recombinant IL-6 from R&D Systems; and Matrigel from BD Pharmingen.

### Cell culture transfection

Cells lines MCF-7, MDA-MB-231, MDA-MB-468, MDA-MB-435, MDA-MB-453, ZR-75, T47D, SKBR-3, BT-474, BT-549, HBL100, SUM159, B16, NIH3T3, and 293T were obtained from the American Type Culture Collection (Manassas, VA) and grown in Dulbecco's modified Eagle's medium (DMEM) or RIPA-1640 medium (Life Technologies) supplemented with 10% fetal bovine serum (FBS; Hyclone) and 1% penicillin/streptomycin (Life Technologies). Cell line MCF-10A was purchased from ATCC and cultured in DMEM/F12 medium (Life Technologies) supplemented with 5% horse serum (Life Technologies), 100 ng/mL cholera toxin (Sigma), 0.5 µg/mL hydrocortisone (Sigma), 10 µg/mL insulin (Sigma), 20 ng/mL epidermal growth factor (R&D), and 1% penicillin/streptomycin. Mycoplasma infection was tested regularly with a PCR-based kit (MP0040, Sigma). All cells lines were incubated at 37 °C in a humidified 5% CO_2_ atmosphere. Transfection of the plasmids and siRNAs into the cells was conducted using Lipofectamine 2000 reagent (Life Technologies) according to the manufacturer’s protocol.

### Western blot and co-immunoprecipitation

For western blotting, cell lysates were prepared as described previously^62^. Protein concentration was measured using Bio-Rad protein assay kit (Hercules, CA) according to the manufacturer’s protocol. Equal amounts of protein were separated by sodium dodecyl sulfate–polyacrylamide gel electrophoresis (SDS-PAGE) and transferred to pre-activated polyvinylidene difluoride membranes (Millipore). The blotted membranes were blocked with 5% fat-free milk for 1 h at room temperature and then incubated overnight at 4 °C with a specific primary antibody. After incubation with HRP-conjugated secondary antibodies for 1.5 h at room temperature, signals were visualized using the ECL chemiluminescence kit (Pierce). For IP, cells were washed with ice-cold phosphate-buffered saline (PBS), pelleted and lysed in IP lysis buffer (Pierce) for 20 min on ice. Cells were then centrifuged at 15,000 × *g* for 10 min at 4 °C. The supernatant was recovered and incubated overnight at 4 °C with the corresponding primary antibody. Protein G Sepharose beads(Amersham/GE) were added and further incubated for 4 °C; the beads were washed three times with PBS and beads boiled for 10 min in 100 µL of 2× SDS loading buffer^63^. Representative original images of immunoblotting results for Figs. 1–6 are shown in Supplementary Fig. 10.

### Luciferase assay

Luciferase assays were performed by co-transfection of the STAT3 luciferase reporter hSIE/APRE and the expression plasmids or siRNAs indicated in the figure legends. In all experiments, pRL-TK vector (Promega, Madison,WI) was transfected as an internal control. Transfected cells were treated with or without IL-6 for STAT3 activation for 6 h. The luciferase activities of the reporters were measured using the Dual Luciferase Reporter Assay System (Promega) according to the manufacturer’s protocol.

### mRNA-seq and real-time quantitative PCR

For the mRNA-seq assay, samples were submitted to Shanghai Biotechnology Corporation for RNA-seq. Poly (A) RNA was purified from total RNA, then converted to double-stranded cDNA; the resulting cDNA samples were sequenced using the standard Solexa protocols. The sequencing reads were mapped to the human genome using tophat (version: 1.0.13). Avadis NGS (version: 1.3) was used to calculate reads per kilobase per million mapped reads (RPKM) values. Differentially expressed genes were called at twofold changes using RPKM. GO and KEGG analyses were performed with DAVID (Database for Annotation, Visualization and Integrated Discovery; http://david.abcc.ncifcrf.gov/). For real-time PCR, total RNA was isolated using Trizol reagent (Invitrogen), then cDNA was generated by reverse transcription of aliquots of RNA using the Takara PrimeScript RT Reagent Kit (Takara) according to the manufacturer’s instruction. The resulting cDNA was used for real-time PCR with SYBR® Premix Ex Taq™ Kit(Takara) in a StepOne Real-Time PCR Detection System (Life Technologies). All expression data were normalized to β-actin-encoding transcript levels. Primers used for real-time PCR are shown in Supplementary Table 2.

### MTT proliferation assay

Cells were seeded at 4000 cells per well in 96-well plates in triplicate, and incubated for indicated time points. Then, 20 µL MTT (Thiazolyl Blue Tetrazolium Bromide, 5 mg/mL, Sigma) was added to each well and plates were incubated for 6 h at 37 °C. The supernatant was aspirated and dimethyl sulfoxide (Sigma) was added 100 µL per well and the plates were incubated for 30 min at 37 °C. The absorbance was measured at 570 nm in a SpectraMax 190 microplate reader (Molecular Devices, USA).

### 3D cell culture

Aliquots of 4000 cells were seeded into each well of 8-well chamber slides coated with 70 μL per well of Matrigel; fresh medium containing Matrigel was added every 3 days. Phase images were captured on an IX51 Microscope (Olympus).

### Cell migration assay

The cell migration assay was performed using modified Boyden chambers in 24-well dishes with Transwell filter inserts provided with 8 µm pore membranes (Corning Inc., Corning, NY, USA). Aliquots of 4 × 10^4^ cells were seeded into each upper chamber of the insert in serum-free medium, and complete medium was added to the lower chamber. After 12 or 24 h, cells were fixed with 4% paraformaldehyde and stained using 0.1% crystal violet. Cells in the upper chamber were carefully removed, and the cells that migrated through the lower side of the filter were imaged (Olympus IX81) and quantified with ImageJ.

### Soft agar assay

Aliquots of 3 × 10^3^ of NIH3T3 v-SRC, NIH3T3 STAT3C, or B16 cells were inoculated into 0.3% agar containing 1× DMEM and seeded in each well of a 6-well plate containing 0.6% agar in 1× DMEM. Cells were grown at 37 °C for 21 days and stained with crystal violet (0.5% w/v).

### Detection of tumor-derived cytokines using antibody arrays

The Human Cytokine Antibody Arrays C5 kit (Raybiotech) was used according to the manufacturer’s instructions. Briefly, each array was blocked and incubated overnight at 4 °C with 1 mL of undiluted condition medium (CM). Samples were aspirated; the array was washed five times, incubated with biotin-conjugated antibodies (1:250) for 2 h at room temperature, and then incubated with HRP-linked secondary antibody (1:1000) for 2 h at room temperature. The membranes were incubated with chemiluminescent substrate and imaged within 10–15 min, since chemiluminescent signals fade over time.

### Detection of human IL-6 concentration by ELISA

Human IL-6 ELISA Kit (Raybiotech) was used according to the manufacturer’s instructions. Briefly, an aliquot (100 µL) of standard or sample was added to each well, and plates were incubated for 2.5 h at room temperature. An aliquot of 100 µL of prepared biotin antibody was added to each well and plates were incubated for 1 h at room temperature. An aliquot of 100 µL of prepared streptavidin solution was added to each well and plates were incubated for 45 min at room temperature. An aliquot of 100 µL of TMB One-Step Substrate Reagent was added to each well and plates were incubated for 30 min at room temperature. Finally, an aliquot of 50 µL of Stop Solution was added to each well and plates were read immediately by measuring absorbance at 450 nm.

### Electrophoretic mobility shift assay

EMSA was performed using the LightShift Chemiluminescent EMSA Kit (Thermo) according to the manufacturer’s protocols. The biotin-conjugated human high-affinity sis inducible element (hSIE) (5′-AGCTTCATTT CCCGTAAATC CCTAAAGCT-3′) was synthesized at Beyotime company; upon receipt, the hSIE was denatured and annealed to form a double strand. Briefly, for the EMSA, nuclear extracts were incubated with biotin-labeled oligonucleotide probe in binding buffer (50% glycerol, 100 mM MgCl2, 1% NP-40, 1 µg/μL poly (dI•dC), 100 mM Tris, 500 mM KCl, 10 mM DTT) for 20 min at room temperature. The DNA–protein complexes were resolved by electrophoresis on 5% non-denaturing polyacrylamide gel in 0.5× TBE buffer at 100 V for 1.5 h, then transferred to a nylon membrane at 380 mA (~100 V) for 30 min. The membrane was crosslinked, blocked in blocking buffer for 15 min, and incubated with HRP-linked streptavidin (1:300) for 15 min with gentle shaking. The membranes were incubated with chemiluminescent substrate and exposed to X-ray film for 2–5 min.

### ChIP assay

ChIP was performed using the ChIP assay kit (Millipore) according to the manufacturer’s instructions. Anti-STAT3 antibody (Cell Signaling Technology) or normal rabbit serum was incubated overnight with sonicated cell lysates. Purified immunoprecipitated DNA was used for quantitative reverse transcription-PCR. Searching of candidate STAT3 binding sites within the *IL-6* promoter region was done by JASPAR database. Primers for ChIP-PCR are shown in Supplementary Table 2.

### Oncomine, GEO data analysis, Kaplan–Meier-Plotter analysis

Oncomine data analyses were performed as previously described^64^. Briefly, we searched for *Wwox* using the following threshold values: fold change of 2, *p* value of 0.05, and gene rank in the top 10% among all differentially expressed genes. All the datasets listed in Oncomine were ordered by *p* value; the values of each published dataset were then linked to the graphical representations of the original microarray dataset. We searched the value of Wwox from the original datasets downloaded from Gene Expression Omnibus (GEO) profiles, and each sample value was log2 transformed. The *p* value was calculated using Student’s *t*-test. To analyze the prognostic value of Wwox in breast cancer, data were analyzed with the Kaplan–Meier (KM) plotter (http://kmplot.com/analysis/). Survival curves were generated using the KM plotter online tool based on data stratified based on the best cut-off. Hazard ratios and *p* values (log-rank *p*) are shown at the top of the panel^65^.

### Tumor samples and IHC analysis

This study was approved by the Institutional Review Board of the Institute for Nutritional Sciences, Chinese Academy of Sciences. Three validated cohorts of patients with informed written consent were used in the study. Specimens were obtained as tissue microarray (TMA) chips from the Biobank Center of the National Engineering Center for Biochip at Shanghai (also known as Shanghai Outdo Biotech Company, Ltd). The TMA specimens were used for IHC analysis. The first cohort, which consisted of 30 cases, was used for IHC score analysis. The second cohort, which consisted of 90 cases, was used for correlation analysis. The third cohort, which consisted of 150 cases, was used for Kaplan–Meier survival analysis. All the patient information for specimens incorporated into the TMA chips are summarized in Supplementary data 1. TMA chips were deparaffinized in xylene, rehydrated with graded ethanol, and washed in dH_2_O. Antigen retrieval was performed in sodium citrate buffer (pH 6.0). After quenching of endogenous peroxidase activity and blocking of nonspecific binding, sections were incubated with primary antibody at 4 °C overnight. After washing with PBS, sections were incubated with the corresponding secondary antibody for 1 h at room temperature. After further washing with PBS, sections were incubated with StrepABComplex/horseradish peroxidase (Dako, Inc., USA) for 1 h at room temperature. Chromogenic immunolocalization was conducted using 0.05% 3,3’-diaminobenzidine (Dako). All sections were counterstained with hematoxylin. Normal serum was used in the place of primary antibody as a negative control.

The IHC evaluation of protein expression intensity in normal adjacent tissues and paired BC tissues was performed independently by two pathologists from the Department of Pathology, Zhongshan Hospital (Fudan University). The pathologists were blinded to the patient’s clinical features; in cases of disagreement, a consensus was reached by joint review. Scoring was as follows: 0, no positive cells detectable; 1, very few positive cells (<5%) showing weak light-brown staining; 2, a few positive cells (<10%) showing weak to medium brown staining; 3, a few positive cells (<10%) showing medium to dark brown staining; 4, large number of positive cells (>10%) showing medium to dark brown staining; 5, large number of positive cells (>20%) exhibiting medium to dark brown staining. When evaluating the expression of proteins in normal tissues and paired cancer tissues, 'high’ means that the score of cancer tissues is higher than the score of the paired normal tissues; in converse, ‘low’ means that the score of cancer tissues is lower than the score of the paired normal tissues. When evaluating the intratumor expression of Wwox and p-STAT3, we defined a score of 0–2 as low and 3–5 as high, respectively^62^.

### Tumor xenografts

All animal experiments were performed in accordance with a protocol approved by the Institutional Animal Care and Use Committee of the Institute for Nutritional Sciences, the Shanghai Institutes for Biologic Sciences, and the Chinese Academy of Sciences. The 4-week-old female nude mice (BALB/c, nu/nu) and 6-week-old C57BL/6J were purchased from the Shanghai Experimental Animal Center (Shanghai, China) and maintained under pathogen-free conditions. On day 0, nude mice were injected subcutaneously on the right flank with tumor cells (1 × 10^6^ cells/mouse) suspended in 100 μL serum-free medium and mixed 1:1 (v/v) with Matrigel. In-life tumor growth was monitored every other day by measuring tumor dimensions (length (L) and width (W)) using calipers. Tumor volume was calculated using the formula for the volume of an ellipsoid sphere (volume = W^2^ x L x 0.5). At the time of killing, tumors were harvested, photographed, and weighed. For the lung metastasis model, 2 × 10^5^ of B16 cells suspended in 100 µL of PBS were injected into 6-week-old C57BL/6J mice via the tail vein. Mice were killed after 21 days, and metastatic nodules on the lungs were counted.

### Statistical analysis

All in vitro experiments were repeated at least three times, and all in vivo experiments were performed at least twice. Two-tailed Student’s *t*-tests were conducted using Prism software, version 5.0 (GraphPad, San Diego, CA). The *p* values of less than 0.05 were considered significant.

### Data availability

Gene expression data have been deposited in the GEO profiles database under the accession codes GSE110810. The authors declare that all the other data supporting the findings of this study are available within the article and its Supplementary Information files and from the corresponding author upon reasonable request.

## Electronic supplementary material

Supplementary Information

Description of Additional Supplementary Files

Supplementary Data 1
