# Supplementary material for: Loss of Wwox drives metastasis in triple-negative breast cancer by JAK2/STAT3 axis
Source: Nat Commun. 2018 Aug 28;9:3486. doi: 10.1038/s41467-018-05852-8 (PMC6113304; doi:10.1038/s41467-018-05852-8)
Supplement: Supplementary file 1 — Supplementary Information [file 41467_2018_5852_MOESM1_ESM.pdf]

Supplementary Information

Loss of Wwox drives metastasis in triple-negative breast cancer by JAK2/STAT3 axis

Chang et al.

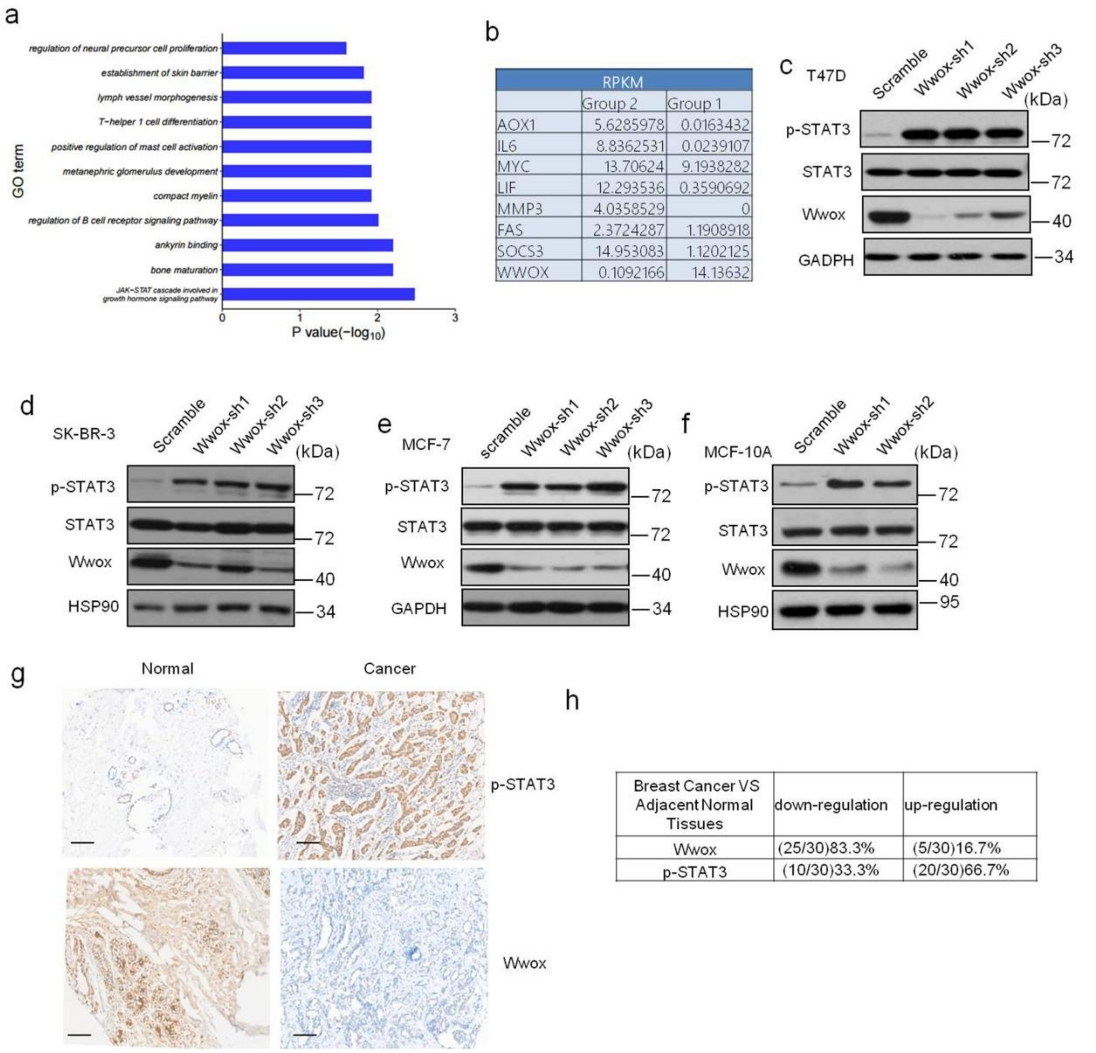

Supplementary Figure 1. Wwox and p-STAT3 are reversely expressed in breast cancer cells. (**a**) GO biological process analysis of deregulated genes in basal cells compared with luminal cells. (**b**) The RPKM value of upregulated genes related to JAK/STAT signaling pathway in luminal cells and basal cells. (**c**-**f**) the protein levels of Wwox and p-STAT3 were examined by Western blotting in T47D (**c**), SK-BR-3 (**d**), MCF-7 (**e**) and MCF-10A(**f**) breast cancer cell lines stably downregulated Wwox expression by Wwox shRNAs. (**g**,**h**) IHC determination of Wwox expression in adjacent normal and paired BC tissues. Representative IHC staining images of Wwox and p-STAT3 (**g**); Results are tabulated as shown (**h**). Scale bar, 100 μm.

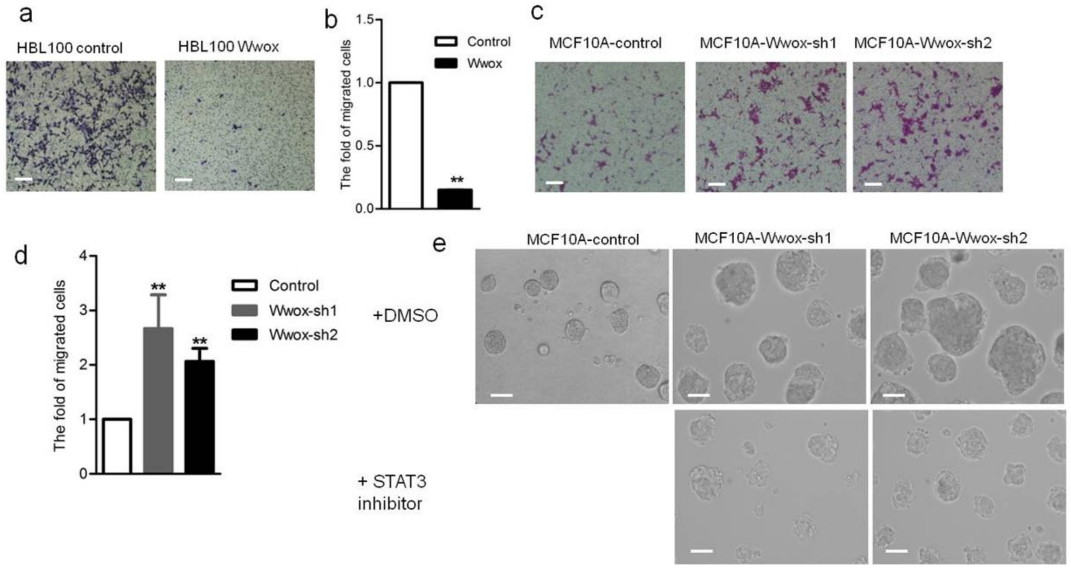

Supplementary Figure 2. Wwox inhibits metastasis in vitro. (**a**,**b**)Transwell migration assay was performed in HBL100 cells stably transfected with control or Wwox-encoding vectors. (**a**) Representative images of migrated HBL100. (**b**) Quantitative results are illustrated for migration in panel A. Data represent the mean ± SD (n=3) from three independent experiments. (**c**, **d**) Transwell migration assay was performed in control or Wwox shRNA-expressing MCF-10A. (**c**) Representative images of migrated MCF-10A, (**d**) Quantitative results are illustrated for migration in panel C. Data represent the mean ± SD (n=3) from three independent experiments. (**e**) Phase-contrast images of MCF-10A cells stably expressing control (CT) or either of two Wwox shRNAs which were treated with DMSO or Stat3 inhibitor Stattic and grown 3D cultures (Day 8). ***p* <0.01, Student's *t*-test. Scale bar, 100 μm.

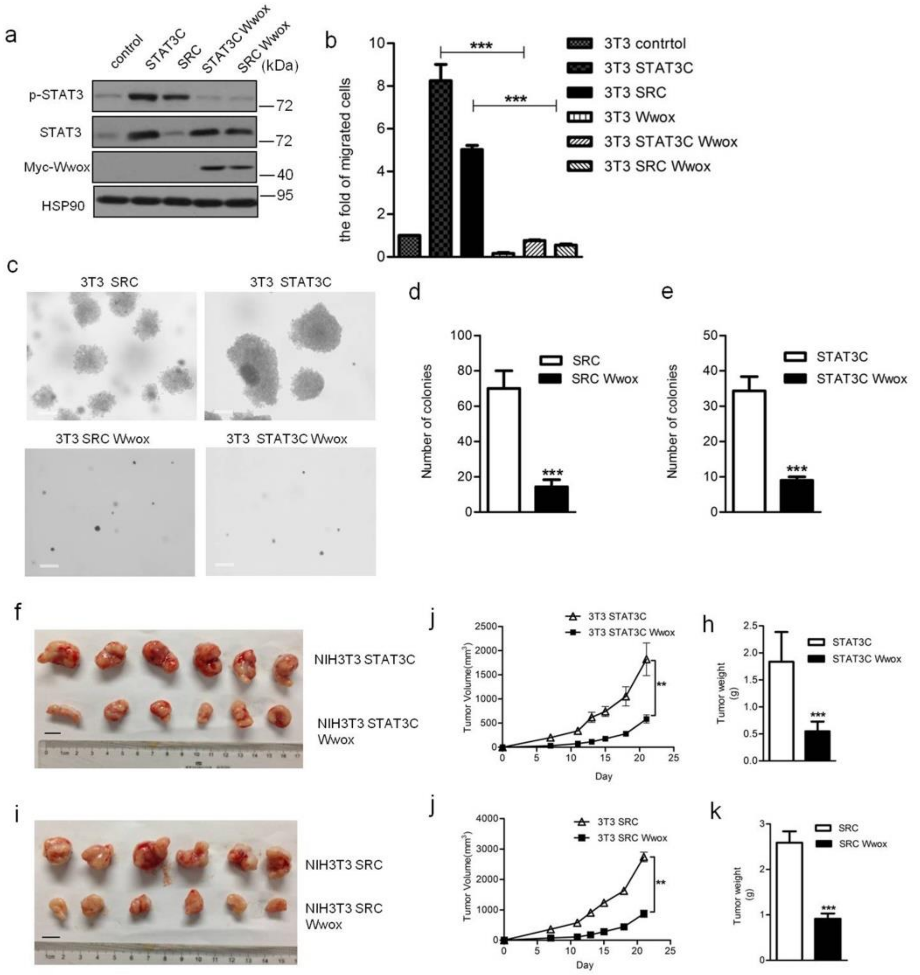

Supplementary Figure 3. Wwox inhibits activated STAT3-induced cell migration and growth. (**a**) Wwox decreases p-STAT3 levels in STAT3C or v-SRC-transformed NIH 3T3 cells. NIH 3T3 cells were transformed with v-SRC (NIH 3T3 v-SRC) or STAT3C (NIH3T3 STAT3C) and further stably transfected with Myc-Wwox (NIH 3T3 v-SRC+Wwox or NIH 3T3 STAT3C+Wwox). (**b**) Wwox inhibits activated STAT3-induced cell migration. Transwell migration assay was performed in different NIH3T3 cell lines. Quantitative results are illustrated for migration. Data represent the mean ± SD (n=3) from three independent experiments. ****p* <0.001.Student's *t*-test. (**c**-**e**) Wwox inhibits p-STAT3-dependent colony formation. $3X10^3$ of NIH 3T3 cells from different lines were

seeded in soft agar in a 6-well plate. After 2 weeks, the colonies were photographed (**c**), and colony numbers were counted in each well in six 40Xmicroscopic fields (**d**, **e**). Results shown are mean ± SD; n = 15, ***p <0.001.Student's t test. Scale bar, 100 μm. (**f**-**k**) Wwox inhibits tumor growth of hyperactivated STAT3 transformed cells. Cells ($5x10^6$) from different lines of NIH3T3 transformed with STAT3C- or v-SRC-encoding constructs were injected into the flanks of four-week-old nude mice. Tumor sizes were monitored over a period of 14 days. For the animals implanted with STAT3C-transformed cells, tumors were harvested, photographed (**f**), and weighed (**h**) at necropsy. In-life tumor sizes are plotted in (**j**). For the animals implanted with v-SRC-transformed cells, tumors were harvested, photographed (**i**), and weighed (**k**) at necropsy. In-life tumor sizes are plotted in (**j**). Results are presented as mean ± SD (n=6) of calculated tumor volume and tumor weight. **p <0.01, ***p <0.001, Student's *t*-test. Scale bar, 1cm.

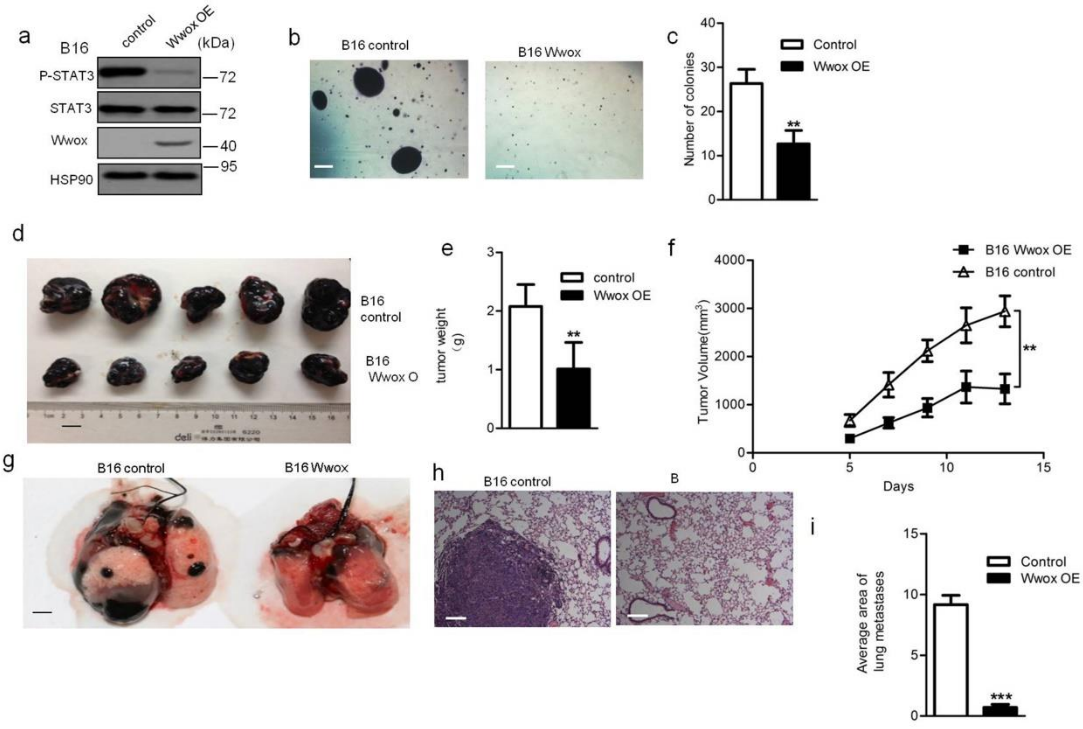

Supplementary Figure 4. Wwox inhibits metastasis in B16 cells. (**a**) Western blot analysis of Wwox and p-STAT3 expression in B16 cells stably transfected with control (CT) or Wwox-encoding vectors. (**b**, **c**) Wwox inhibits B16 cells colony formation in soft agar. 3×10^3 cells were seeded in a 6-well plate. After 2 weeks, the colony number was counted in each well in six 40Xmicroscopic fields. An image (**b**) and a quantitative presentation (**c**) of the colonies are shown. Statistical analyses were performed using Student's *t*-test. Results shown are mean± SD(n=6), **p <0.01. Scale bar, 100 μm. (**d**-**f**) Wwox represses the tumor growth. Four-week-old nude mice were subcutaneously injected with $5x10^5$ of mock B16 and Wwox overexpression B16 cells. Tumor sizes were monitored during a period of 14 days (**f**). At autopsy, tumors were harvested, photographed (**d**) and weighed (**e**). Results are presented as mean± SD (n=5) of calculated tumor weight. **p <0.01, Student's *t*-test. Scale bar, 1cm. (**g**-**i**) Ten-week-old C57BL/6J mice were injected into the tail vein with the indicated lines of B16 cells. After 21days, mice were euthanized, and

representative metastatic nodules on lungs were shown (**g**), Scale bar, 1cm. Lung tissues were fixed and stained with haematoxylin and eosin (H&E) (**h**), Scale bar, 100 μm. The average area of metastatic lesions in each specimen was counted (**i**). Results are presented as mean± SD (n=6). ***p <0.001, Student's *t*-test.

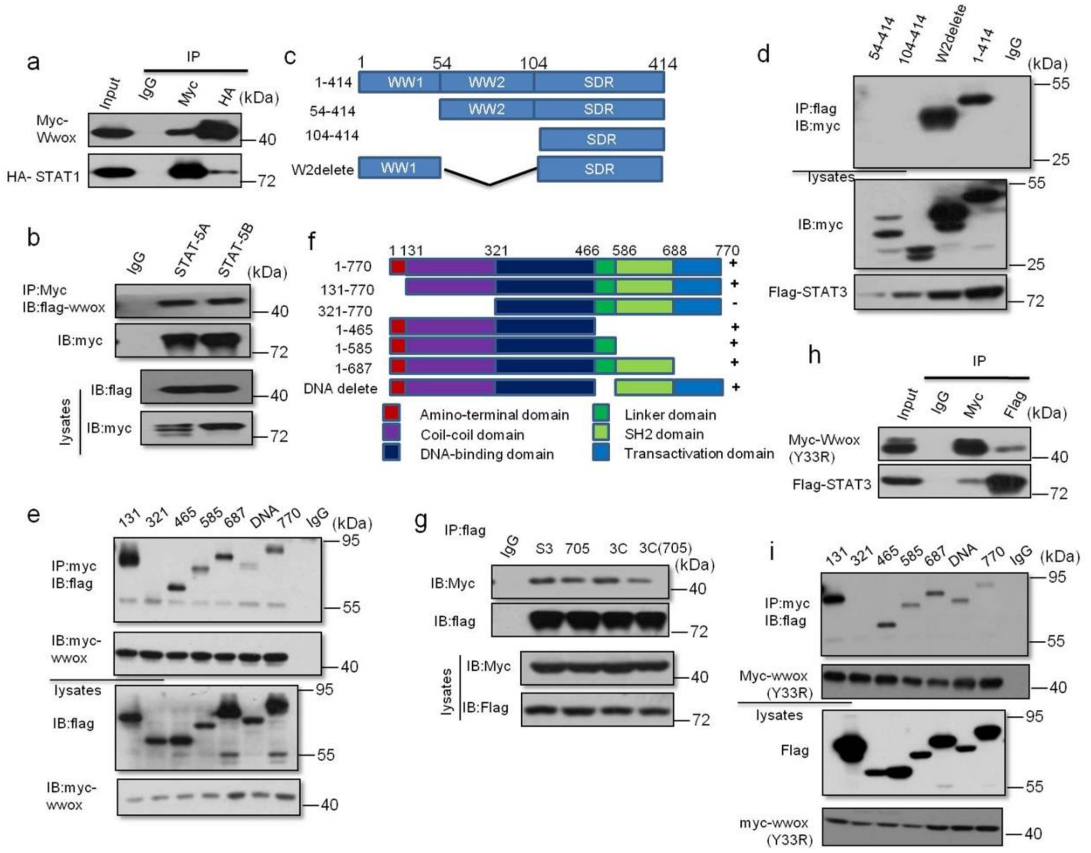

Supplementary Figure 5. STAT3 interacts with Wwox. (**a**) Wwox interacts with STAT1. 293T cells were transfected with plasmids expressing Myc-tagged full-size Wwox or HA-tagged full-length STAT1 as indicated. Whole-cell lysates were used in immunoprecipitation (IP) with anti-Myc antibody or anti-Flag M2 antibody. (**b**) Wwox interacts with STAT5. Myc-Wwox was coexpressed with Flag-tagged STAT5A or STAT5B in 293T cells for IP with an anti-Myc antibody. (**c**, **d**) Wwox interacts with STAT3 mainly through WW1 domain. Schematic representation of full-length Wwox and its deletion mutants(**c**). Immunoprecipitation and immunoblot of cell lysates from HEK293 cells expressing Flag-tagged STAT3 and Myc-tagged Wwox or its deletion mutants. Whole cell lysates were immunoprecipitated with anti-Flag(M2) and immunoblotted with anti-Myc (**d**). (**e**, **f**) Wwox interacts with the Coil-coil domain of STAT3.Schematic representation of

full-length STAT3 and its deletion mutants(**f**). Immunoprecipitation and immunoblot of cell lysates from HEK293 cells expressing Myc-tagged Wwox and Flag-tagged STAT3 or its deletion mutants. Whole cell lysates were immunoprecipitated with anti-Myc and immunoblotted with anti-Flag(**e**).(**g**) An interaction pattern of Wwox with different STAT3 mutations. STAT3C (indicated as 3C), STAT3 (Y705F) (indicated as 705) and mutant of STAT3C at Y705 (indicated as 3C (705)), which maintains dimerization but lost phosphoryation at Y705F, were coexpressed with Myc-Wwox in 293T cells for the IP experiment.. (**h**) Mutant Wwox (Y33R) interacts with STAT3. 293T cells were transfected with the indicated constructs and cell lysates were used for IP experiments. (**i**) Wwox interacts with STAT3 via its Coil-coil domain. Wwox was co-expressed with different STAT3 domains in 293T cells. IP was performed with an anti-Flag antibody and precipitants were immunoblotted with an anti-Myc antibody. Data are representative of two independent experiments.

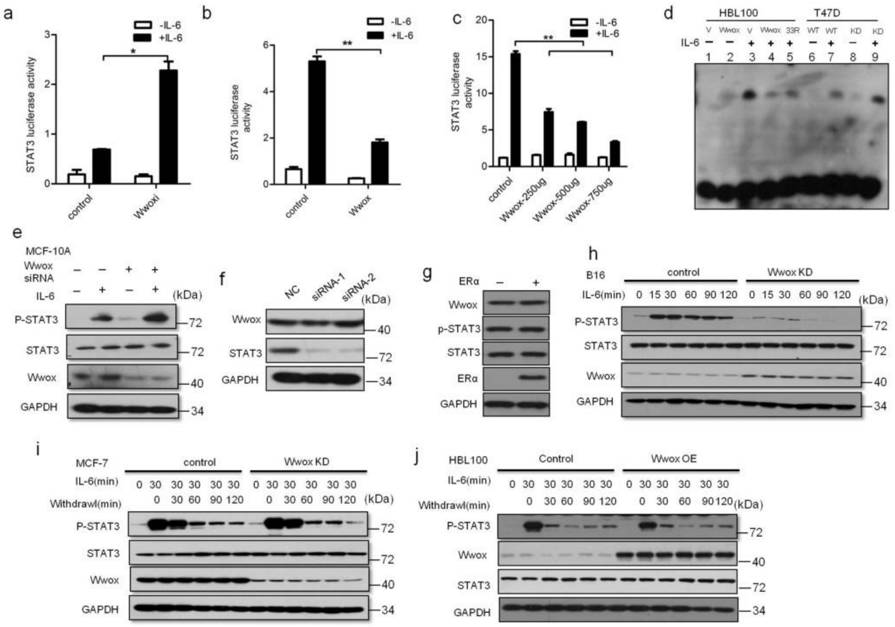

Supplementary Figure 6. Wwox inhibits STAT3 transcription activity by decreasing the level of p-STAT3. (**a**, **b**) Wwox inhibits STAT3 transcriptional activity. Cells were cotransfected with APRE luciferase reporter and *Renilla* luciferase reporter, with depletion of Wwox (Wwoxi) in T47D cells (**a**) or overexpression of Wwox (Wwox) in 293T cells(**b**). Relative luciferase activity data are presented as mean ± SD from three independent experiments. *p <0.05, **p <0.01. (**c**) Wwox inhibits STAT3 transcriptional activity in a dose-dependent manner. 293T cells were transfected with APRE luciferase reporter and *Renilla* luciferase reporter in combination with increasing amounts of Wwox expression vectors as indicated. Twenty-four hours later, cell extracts were prepared and luciferase activities quantified. (**d**) Wwox decreases the DNA binding ability of STAT3. EMSA assays were performed using a biotin-labeled high-affinity binding site for STAT3. HBL100 cells were transfected with empty vector (V), Wwox, and Wwox (Y33R) mutant and T47D cells were transfected with Wwox siRNA. HBL100 or T47D cells were treated with or without

IL6 for 30min. (**e**) Wwox reduces p-STAT3 levels. MCF-10A cells were transfected with Wwox siRNA and were treated with IL-6 for 30 min. The whole cell lysates were immunoblotted with the indicated antibodies. (**f**) The protein levels of Wwox were examined in MCF-7 cells, which were transfected with STAT3 siRNAs. (**g**) MCF-10A cells were transfected with ER plasmid. The whole cell lysates were immunoblotted with the indicated antibodies. (**h**) Wwox inhibits STAT3 phoshorylation. Cells were treated with IL6 for different time points when Wwox was overexpressed in B16 cells. Phosphorylated and total STAT3 levels were examined. (**i**, **j**) Wwox has no effect on STAT3 dephosphorylation. MCF-7 Cells or HBL100 were treated with IL-6 and then subjected to starvation (indicated as withdrawal). The whole cell lysates were immunoblotted with the indicated antibodies.

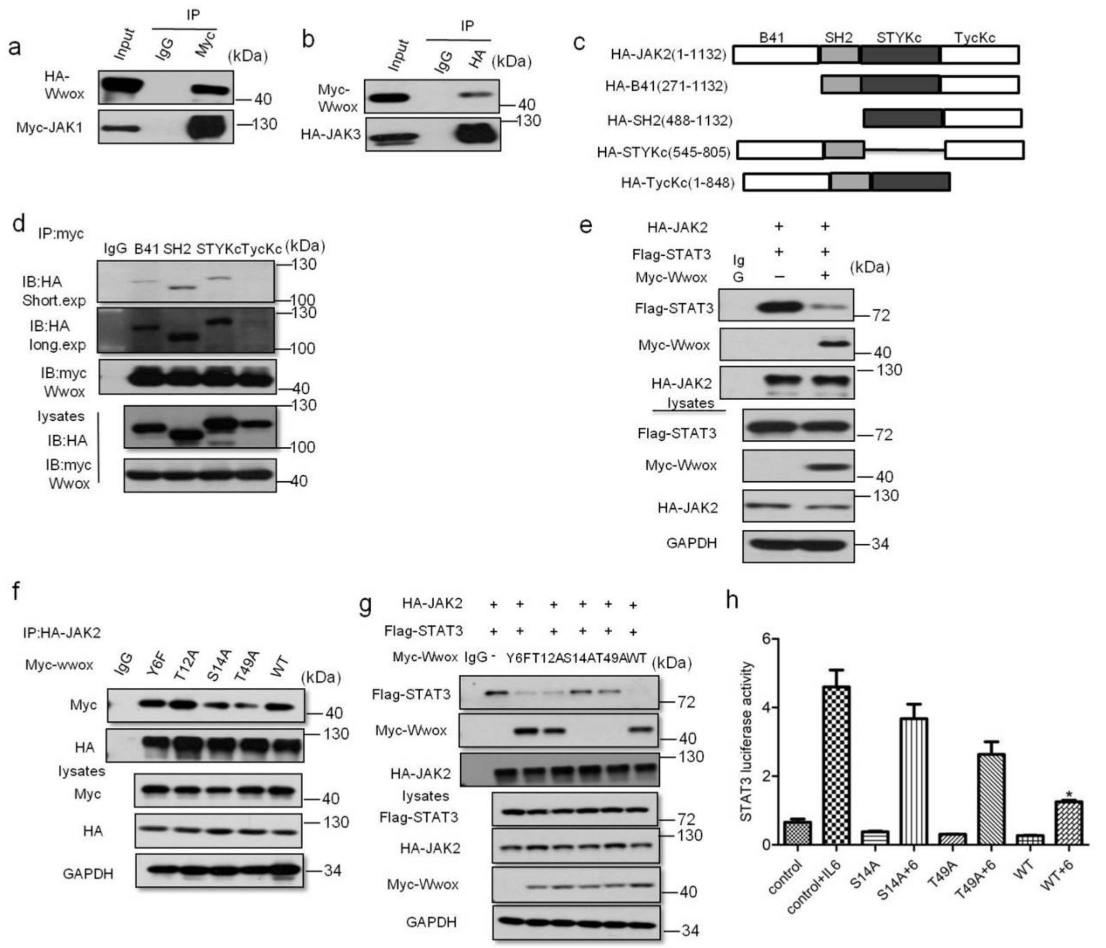

Supplementary Figure 7. Wwox attenuates the interaction between JAK2 and STAT3. (**a**) Over-expressed HA-Wwox interacts with Myc-JAK1 in 293T cells. 293T cells were transfected with Myc-JAK1 and HA-Wwox. Immunoprecipitation (IP) was performed with an anti-Myc antibody. (**b**) Myc-Wwox interacts with HA-JAK3 in 293T cells. 293T cells were transfected with Myc-Wwox and HA-JAK3 Immunoprecipitation (IP) was performed with an anti-HA antibody. (**c**, **d**) Wwox interacts with JAK2 via its TycKc domain. Wwox was co-expressed with different JAK2 domains in 293T cells. (**c**) Schematic representation of full-length JAK2 and its deletion mutants. (**d**) Whole cell lysates were immunoprecipitated with anti-Myc and immunoblotted with anti-HA. (**e**) Overexpression of Wwox inhibits the interaction of JAK2 with STAT3. 293T cells were transfected with

constructs encoding HA-JAK2 and Flag-STAT3, along with a Wwox-encoding plasmid; cell lysates were subjected to IP and IB analyses. (**f**) an interaction pattern of JAK2 with different Wwox mutations. The predicted phosphorylation sites of WW1 domain were mutated and were transfected with JAK2 in 293T cells. Lysates were subjected to IP and IB analyses. (**g**) Dynamic changes of JAK2-STAT3 interaction when different Wwox mutations were co-expressed in 293T cells. Lysates of 293T cells were subjected to IP and IB analyses. (**h**) 293T cells were co-transfected with APRE luciferase reporter and Renilla luciferase reporter; with different Wwox mutations. Relative luciferase activity data are presented as mean ± SD from three independent experiments.*$p < 0.05$.

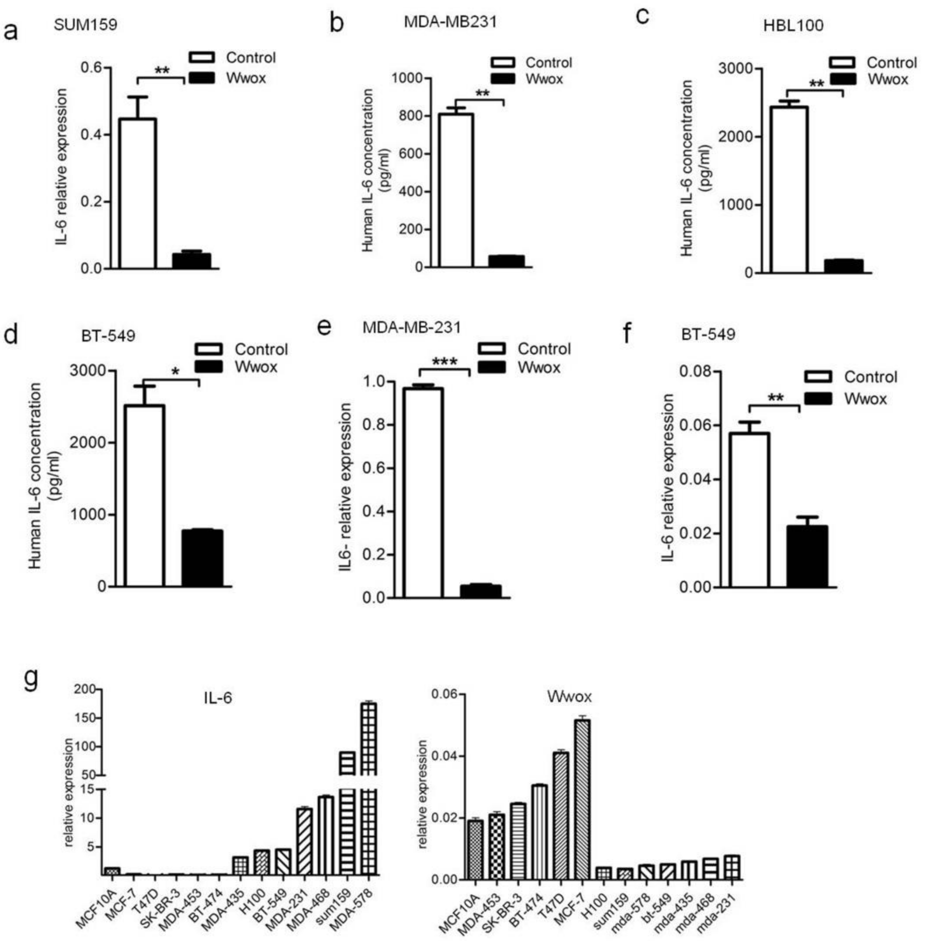

Supplementary Figure 8. Wwox inhibits IL-6 induction. (**a**) Real-time PCR analysis of the mRNA expression of the IL-6 in SUM159. (**b**-**d**) IL-6 production analyzed by ELISA. The cytokine levels in the medium were detected in MDA-MB-231(**b**), HBL100 (**c**) and BT-549 (**d**). *p <0.05, **p <0.01, Student's *t*-test. (**e**,**f**) Real-time PCR analysis of the mRNA expression of the IL6.The mRNA levels were detected in MDA-231(**e**) and BT-549(**f**). (**g**)The mRNA levels of IL6 and Wwox between luminal breast cancer cells and basal-like breast cancer cells were analyzed by Real-time PCR.

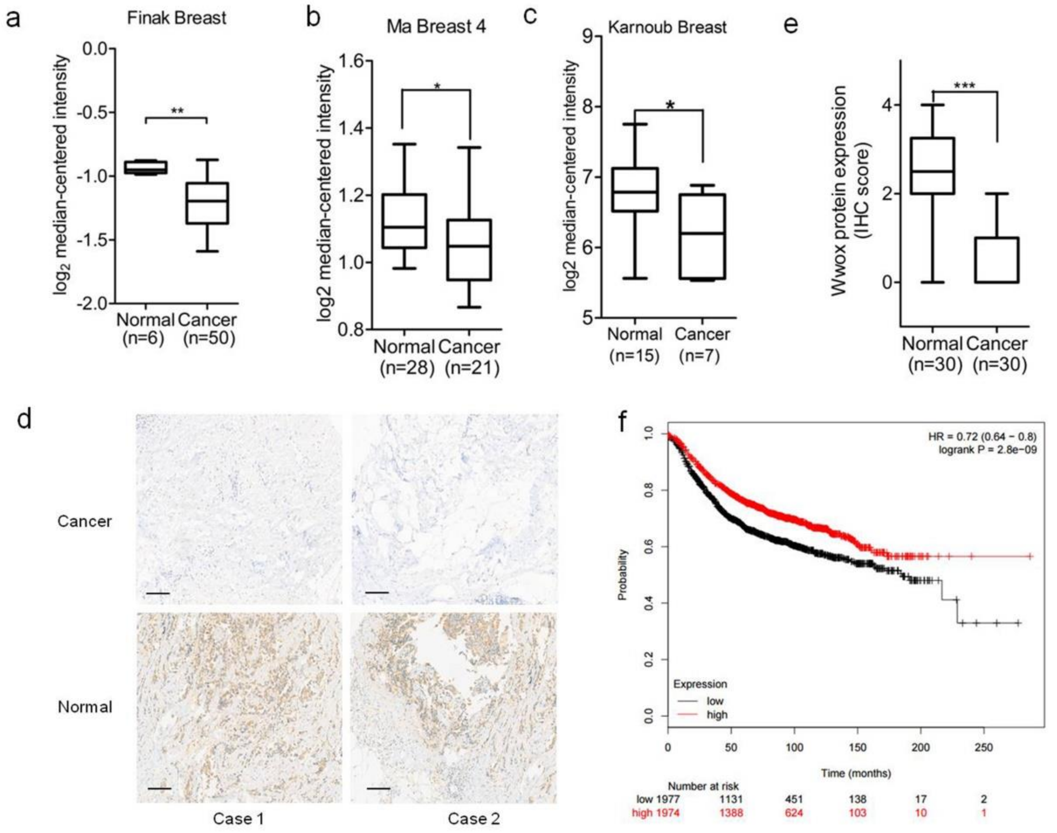

Supplementary Figure 9. The levels of Wwox expression in breast cancer (BC) have prognostic implications. (**a**-**c**) Box plots comparing *Wwox* mRNA levels in human normal breast tissues and BC tissues in published data sets from Oncomine. *p <0.05, Student's *t*-test. (**d**, **e**) IHC staining for Wwox in adjacent normal and paired BC tissues. (**d**). Box plots of Wwox protein expression assessed by blinded IHC analyses of 30 normal and paired BC tissues(**e**). ***p <0.001, Student's *t*-test. Scale bar, 100 µm. (**f**) Kaplan-Meier survival analysis for assessment of Relapse-Free Survival (RFS) in BC patients classified by relative (high or low) tumor Wwox level. Survival curves were generated by using the Kaplan-Meier Plotter online tool based on data stratified based on the best cut-off. Curves were compared by hazard ratios (HR) and p values (log rank p).

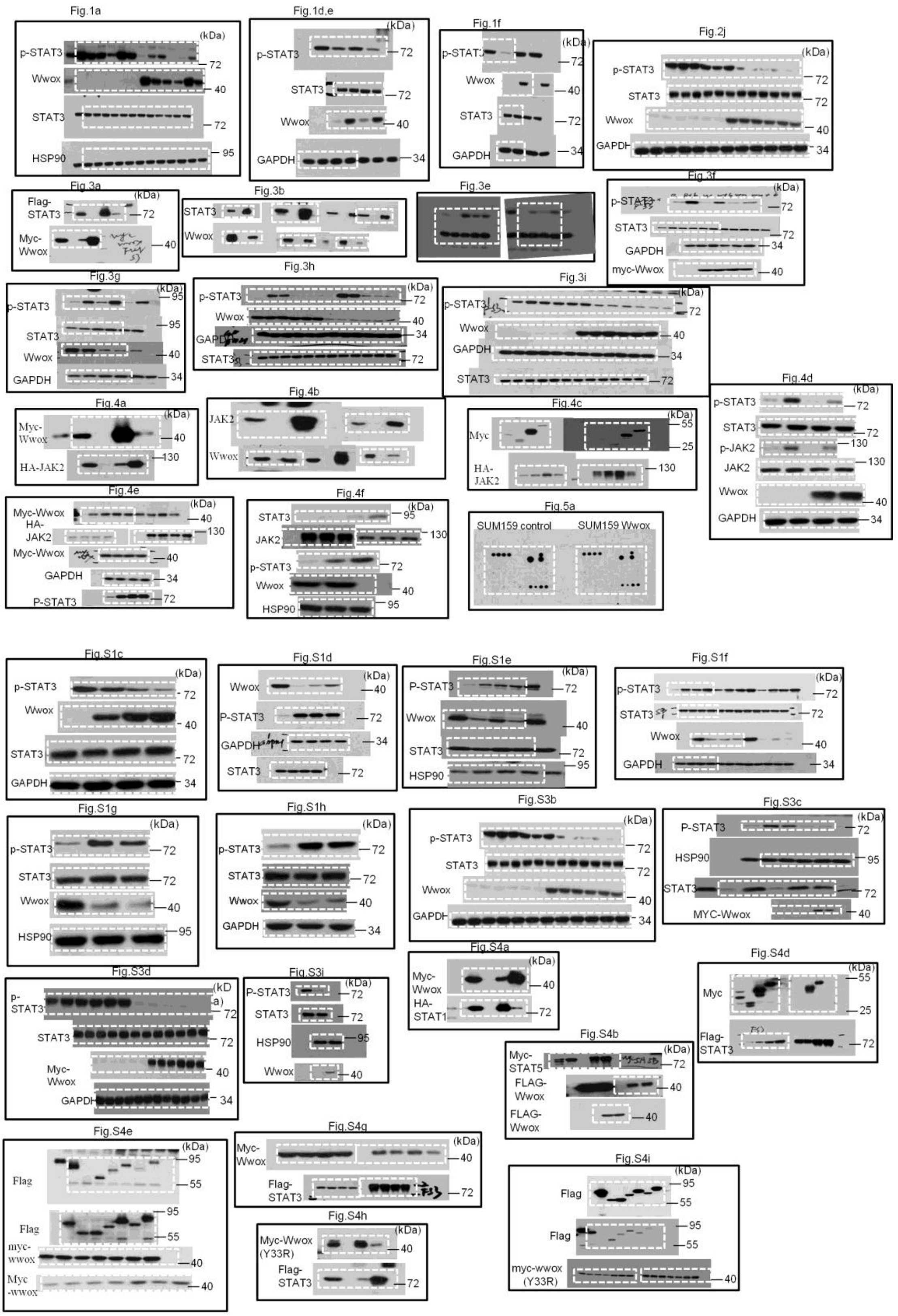
Fig.1a
(kDa)
p-STAT3 — 72
Wwox — 40
STAT3 — 72
HSP90 — 95
Fig.1d,e
(kDa)
p-STAT3 — 72
STAT3 — 72
Wwox — 40
GAPDH — 34
Fig.1f
(kDa)
p-STAT3 — 72
Wwox — 40
STAT3 — 72
GAPDH — 34
Fig.2j
(kDa)
p-STAT3 — 72
STAT3 — 72
Wwox — 40
GAPDH — 34
Fig.3a
(kDa)
Flag-STAT3 — 72
Myc-Wwox — 40
Fig.3b
STAT3
Wwox
Fig.3e
Fig.3f
(kDa)
p-STAT3 — 72
STAT3 — 72
GAPDH — 34
myc-Wwox — 40
Fig.3g
(kDa)
p-STAT3 — 95
STAT3 — 95
Wwox — 40
GAPDH — 34
Fig.3h
(kDa)
p-STAT3 — 72
Wwox — 40
GAPDH — 34
STAT3 — 72
Fig.3i
(kDa)
p-STAT3 — 72
Wwox — 40
GAPDH — 34
STAT3 — 72
Fig.4a
(kDa)
Myc-Wwox — 40
HA-JAK2 — 130
Fig.4b
JAK2
Wwox
Fig.4c
(kDa)
Myc — 55
— 25
HA-JAK2 — 130
Fig.4d
(kDa)
p-STAT3 — 72
STAT3 — 72
p-JAK2 — 130
JAK2 — 130
Wwox — 40
GAPDH — 34
Fig.4e
(kDa)
Myc-Wwox — 40
HA-JAK2 — 130
Myc-Wwox — 40
GAPDH — 34
P-STAT3 — 72
Fig.4f
(kDa)
STAT3 — 95
JAK2 — 130
p-STAT3 — 72
Wwox — 40
HSP90 — 95
Fig.5a
SUM159 control
SUM159 Wwox
Fig.S1c
(kDa)
p-STAT3 — 72
Wwox — 40
STAT3 — 72
GAPDH — 34
Fig.S1d
(kDa)
Wwox — 40
P-STAT3 — 72
GAPDH — 34
STAT3 — 72
Fig.S1e
(kDa)
P-STAT3 — 72
Wwox — 40
STAT3 — 72
HSP90 — 95
Fig.S1f
(kDa)
p-STAT3 — 72
STAT3 — 72
Wwox — 40
GAPDH — 34
Fig.S1g
(kDa)
p-STAT3 — 72
STAT3 — 72
Wwox — 40
HSP90 — 95
Fig.S1h
(kDa)
p-STAT3 — 72
STAT3 — 72
Wwox — 40
GAPDH — 34
Fig.S3b
(kDa)
p-STAT3 — 72
STAT3 — 72
Wwox — 40
GAPDH — 34
Fig.S3c
(kDa)
P-STAT3 — 72
HSP90 — 95
STAT3 — 72
MYC-Wwox — 40
Fig.S3d
(kDa)
p-STAT3 — 72
STAT3 — 72
Myc-Wwox — 40
GAPDH — 34
Fig.S3i
(kDa)
P-STAT3 — 72
STAT3 — 72
HSP90 — 95
Wwox — 40
Fig.S4a
(kDa)
Myc-Wwox — 40
HA-STAT1 — 72
Fig.S4b
(kDa)
Myc-STAT5 — 72
FLAG-Wwox — 40
FLAG-Wwox — 40
Fig.S4d
(kDa)
Myc — 55
— 25
Flag-STAT3 — 72
Fig.S4e
(kDa)
Flag — 95
— 55
Flag — 95
— 55
myc-wwox — 40
Myc-wwox — 40
Fig.S4g
(kDa)
Myc-Wwox — 40
Flag-STAT3 — 72
Fig.S4h
(kDa)
Myc-Wwox (Y33R) — 40
Flag-STAT3 — 72
Fig.S4i
(kDa)
Flag — 95
— 55
Flag — 95
— 55
myc-wwox (Y33R) — 40

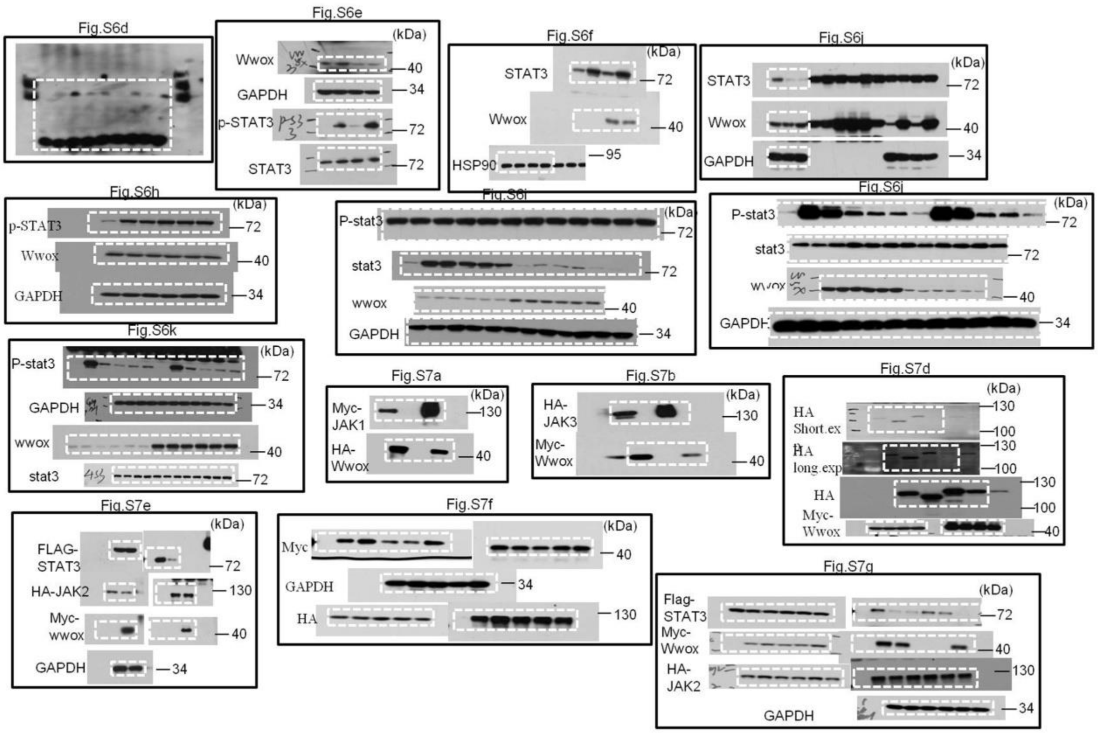

Supplementary Figure 10. Representative original images of immunoblotting results for Figure.1-6 and Fig S1-S9.

Supplementary Tables

Supplementary Table 1. Relationship Between Expression of Wwox in breast Cancer and Clinical Features

**Characteristics**	**Wwox**		***P* value**
	DOWN (83)	UP (67)	
Average Age (years)	54	54.3	0.8810[a]
Average Tumor Size (cm^3)	37.19	16.37	0.01975[a,*]
TNM Stage			
I	3	11	
II	47	39	
III	33	17	0.0121[b,*]
Lymph Node Metastasis			
No	27	28	
Yes	56	39	0.1956[b,*]
Tumor Grade			
I	13	24	
II	69	43	
III	1	0	0.01292[b,*]

a,unpaired Student's t-test; b, χ2 test. *P<0.05, **P<0.01.
TNM:Tumor/Node/Metastasis.

Supplementary Table 2. Primers used for Real-Time PCR and Chip

	Real-Time PCR Primers	
Gene	Forward Primer	Reverse Primer
IL6-F	TCGAGCCCACCGGGAACGAA	GCAACTGGACCGAAGGCGCT
Wwox	GAACATCCAAAAACTGGAAAAAG	CATCAGTTTCTTGTTCCCATCC
BCL-XL	CCCAGAGTTTGAGCCGAGTG	CCCATCCCTTCGTCGTCCT
C-myc	GGCTCCTGGCAAAAGGTCA	CTGCGTAGTTGTGCTGATGT
CyclinD1	GCTGCGAAGTGGAAACCATC	CCTCCTTCTGCACACATTTGAA
β-actin	CCTGCACCCACACAAT	GGGCGGACTCGTCAAC
	Chip-PCR Primers	
	Forward Primer	Reverse Primer
site 1	GAGCACTAAAAATG	TCACAATCGGTTTC
