# Supplementary material for: Loss of Wwox drives metastasis in triple-negative breast cancer by JAK2/STAT3 axis
Source: Nat Commun. 2018 Aug 28;9:3486. doi: 10.1038/s41467-018-05852-8 (PMC6113304; doi:10.1038/s41467-018-05852-8)
Supplement: Supplementary file 2 — Description of Additional Supplementary Files [file 41467_2018_5852_MOESM2_ESM.pdf]

Description of Additional Supplementary Files

File Name: Supplementary Data 1
Description: Clinical summary of three validated cohorts of breast cancer patients
